# Biomechanical consequences of cement discoplasty: An *in vitro* study on thoraco-lumbar human spines

**DOI:** 10.3389/fbioe.2022.1040695

**Published:** 2022-12-02

**Authors:** Chloé Techens, Sara Montanari, Ferenc Bereczki, Peter Endre Eltes, Aron Lazary, Luca Cristofolini

**Affiliations:** ^1^ Department of Industrial Engineering, School of Engineering and Architecture, Alma Mater Studiorum—Università di Bologna, Bologna, Italy; ^2^ In Silico Biomechanics Laboratory, National Center for Spinal Disorders, Buda Health Center, Budapest, Hungary; ^3^ Department of Spinal Surgery, Department of Orthopaedics, Semmelweis University, Budapest, Hungary; ^4^ School of PhD Studies, Semmelweis University, Budapest, Hungary

**Keywords:** percutaneous cement discoplasty (PCD), biomechanics, spine, intervertebral disc, digital image correlation (DIC), intervertebral disc degeneration

## Abstract

With the ageing of the population, there is an increasing need for minimally invasive spine surgeries to relieve pain and improve quality of life. Percutaneous Cement Discoplasty is a minimally invasive technique to treat advanced disc degeneration, including vacuum phenomenon. The present study aimed to develop an *in vitro* model of percutaneous cement discoplasty to investigate its consequences on the spine biomechanics in comparison with the degenerated condition. Human spinal segments (*n* = 27) were tested at 50% body weight in flexion and extension. Posterior disc height, range of motion, segment stiffness, and strains were measured using Digital Image Correlation. The cement distribution was also studied on CT scans. As main result, percutaneous cement discoplasty restored the posterior disc height by 41% for flexion and 35% for extension. Range of motion was significantly reduced only in flexion by 27%, and stiffness increased accordingly. The injected cement volume was 4.56 ± 1.78 ml (mean ± SD). Some specimens (*n* = 7) exhibited cement perforation of one endplate. The thickness of the cement mass moderately correlated with the posterior disc height and range of motion with different trends for flexions vs. extension. Finally, extreme strains on the discs were reduced by percutaneous cement discoplasty, with modified patterns of the distribution. To conclude, this study supported clinical observations in term of recovered disc height close to the foramen, while percutaneous cement discoplasty helped stabilize the spine in flexion and did not increase the risk of tissue damage in the annulus.

## 1 Introduction

The ageing of the global population due to increasing life expectancy (Fuster, 2017) results in the changing epidemiology of disease and spinal disorders (Fehlings et al., 2015). In the ageing spine, the intervertebral disc degeneration (IDD) leads to biomechanical and structural changes of the spine (Kirnaz et al., 2022). The terminal disc degeneration is characterized by total disorganization of the intervertebral tissue, and complete resorption of the nucleus pulposus causing in many cases: this condition is often referred to as “vacuum phenomenon” in the clinical context (Knutsson, 1942; Samuel, 1948; Morishita, et al., 2008). IDD-related structural changes lead to biomechanical malfunctions (Inoue and Espinoza Orías, 2011), such as segmental instability. Surgical treatment possibilities of segmental instability in elderly patients are limited (Fehlings et al., 2015). Minimally invasive surgical (MIS) procedures are the preferred options (Yue et al., 2010). Percutaneous cement discoplasty (PCD) is a MIS procedure, where the vacuum space in the intervertebral disc is filled with percutaneously injected acrylic cement. The PCD procedure is expected to provide a segmental stabilizing effect and indirect decompression of the neuronal elements (Varga et al., 2015; Sola et al., 2018; Kiss et al., 2019). Initially PCD was biomechanically investigated on cervical discs (Roosen, 1982; Wilke, et al., 2000). However, PCD as low-back-pain treatment has only been evaluated in terms of patient outcome by clinical studies (Varga et al., 2015; Kiss et al., 2019; Camino Willhuber et al., 2020). Recently, *in vivo* models of PCD have been developed on porcine and ovine specimens (Techens et al., 2020; Ghandour et al., 2022) proposing promising methods for the experimental evaluation of the surgery biomechanics. In addition, PCD has started to be investigated *in silico*, bringing complementary data on the spine stability and load sharing. A numerical study assessed the impact of injected cement volume and reduction of the nerve root stress following PCD (Jia, et al., 2022). Another study compared the effect of friction at the cement-endplate interface on the disc tissue stress (Li et al., 2022). If PCD appeared efficient to relieve patient’s pain, biomechanics of the human spine following the surgery remain largely unknown and require supplementary investigations (Techens et al., 2022). Combining more experimental and numerical studies would help expanding the research on PCD and improve the surgical technique.This study aims at investigating the consequences of percutaneous cement discoplasty on the biomechanics of the human spine with respect to the pre-operative degenerated condition. Therefore, the first objective was to develop a reliable *in vitro* model of percutaneous cement discoplasty. This was then used to evaluate the *in vitro* biomechanical behaviour of the treated segment. The core objective of this study was monitoring the biomechanical effects of PCD and identifying the potential links between PCD and the biomechanical outcomes in order to assess the benefits and detect potential detrimental effects. In particular, we hypothesized that PCD would increase the disc height in the posterior region with respect to the degenerated condition. We furthermore hypothesized that PCD would impact the intervertebral kinematics. Finally, we conjectured that, as a side effect, PCD could possibly represent a challenge for the surrounding tissue since the cement mass stiffness differs from the nucleus pulposus. We also hypothesized that the cement volume and its distribution inside the disc would impact on the biomechanical behaviour of the treated functional spinal unit (FSU).

## 2 Materials and methods

### 2.1 Compliance with ethical standards

This study was performed in line with the principles of the Declaration of Helsinki. Approval was granted by the Bioethics Committee of the University of Bologna (Prot. 76497, 1 June 2018). The cadaveric spines were obtained through two institutions: an international donation program (International Institute for the Advancement of Medicine) and the hospital of the NCSD after ethical approval of both entities.

### 2.2 Overview of the study

**FIGURE 1:**
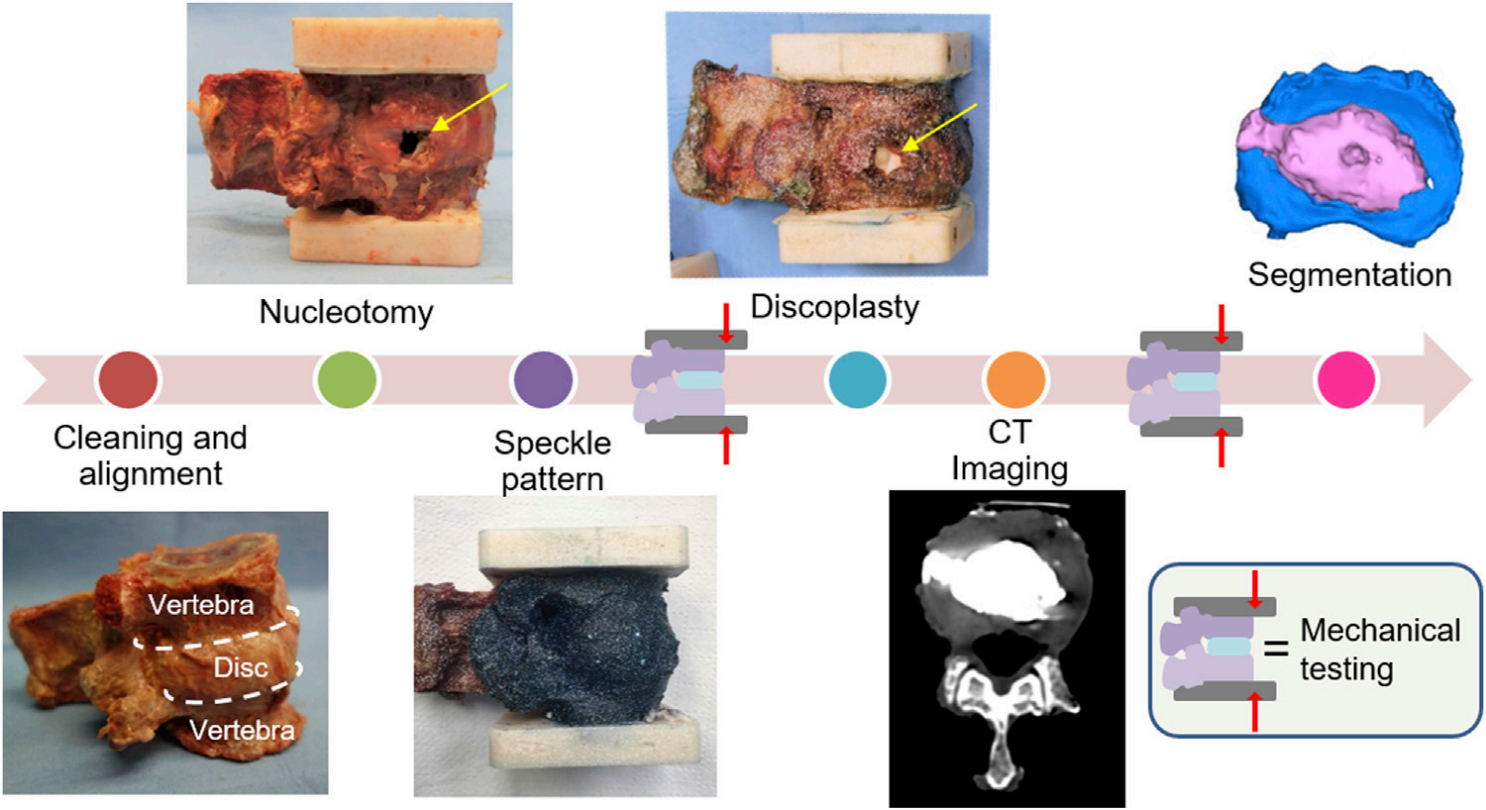
Experimental workflow of the study. The specimens were prepared with a high-contrast speckle pattern to allow measuring displacements and strains under load with digital image correlations. Each specimen underwent biomechanical testing (under the same loading conditions) after nucleotomy and after simulated percutaneous cement discoplasty. The cement injected was investigated on CT images.

PCD is the ultimate treatment for polymorbid patients. This surgery does not aim to completely restore the conditions of a healthy spine, but to mechanically act on the disc foramen to relieve the pain. Thus, this study aimed to assess whether PCD would recover the disc height and impact the intervertebral kinematics in comparison with degenerated discs. The overall workflow is presented in this section; additional technical explanations are detailed in Supplementary Material S1 File. Functional spinal units (FSUs) were prepared for testing (Figure 1). They were biomechanically tested non-destructively after simulating disc degeneration. Then percutaneous cement discoplasty was simulated. The specimens were re-tested under the same loading conditions. Kinematics and strains were measured using digital image correlation (DIC).

### 2.3 Cadaveric specimens

For this study, 27 FSUs were extracted from 15 Caucasian lumbar spines (9 males/6 females) aged 35 to 86 years old. Death was unrelated to a spine disease. Based on computed tomography (CT) scan images, specimens with fractures or bridged osteophytes were excluded from the study by a clinician. Only specimens presenting intact endplates were selected. The selection did not consider the degree of disc degeneration. All soft tissues were carefully removed from the segment preserving the anterior (ALL), supraspinous and posterior ligaments, the facet joints, and the intervertebral disc (IVD) to keep the natural kinematics of the segment (Behrsin and Briggs, 1988; Palanca et al., 2020). As muscles were removed from the spine segments, rigor mortis did not affect the tests. Each segment disc was horizontally aligned, using a six-degree-of-freedom clamp; both segment extremities were embedded with acrylic cement. Specimens were stored at −28°C between cleaning and testing phases, and were thawed in physiological solution at room temperature prior to each test phase; hydration was granted during preparation and testing spraying the specimens (Tan and Uppuganti, 2012).

### 2.4 Surgical procedure

#### 2.4.1 Nucleotomy

PCD is recommended for advanced degeneration of the disc, when a vacuum is observed instead of the nucleus pulposus (NP), inducing a negative pressure within the disc (Varga et al., 2015; Sola et al., 2018). As donor specimens with a vacuum disc are complicated to obtain, a similar disc degeneration state was artificially created by manually emptying the disc. This degenerated disc simulation has been previously developed on animal specimens (Moissonnier et al., 2014; Techens et al., 2020) to provide the anatomical vacuum characteristics needed for PCD using a substitutive method. A rectangular incision as high as the disc and 5–8 mm wide was performed with a scalpel blade in the annulus fibrosus on the lateral side (Figure 1), preferably on the side showing irregularities (small osteophytes, wrinkled tissues). Although it differed from the clinical posterior approach used for PCD, lateral fenestration was chosen in consideration of the loading directions as it avoided damaging the disc and ligaments in the posterior region. The nucleus pulposus was extracted through the excision and the cartilaginous endplates were shaved by scratching the cartilage off by a spine surgeon.

As the incision of the annulus fibrosus (AF) was suspected to critically affect the biomechanics of the remaining annulus, a separate methodological study was performed on eight additional specimens to quantify the consequences of this preparation (Supplementary Material S2 File). Briefly, only NP removal significantly impacted the PDH. AF incision did not significantly impact the posterior disc height nor the biomechanics.

#### 2.4.2 Cement discoplasty

After being tested in a simulated degenerated condition, the specimens were treated with a highly radiopaque acrylic cement (Mendec Spine; Tecres, Sommacampagna, Italy, containing 30% BaSo4). Acrylic cement is known to be biocompatible and so commonly used in other spine surgeries such as vertebroplasty. The cement was prepared as clinically recommended (Sola et al., 2018), mixing the component at room temperature, and waiting a few minutes to obtain the desired viscosity. It was injected inside of the disc through the incision performed during nucleotomy until the cement would fill the cavity (Figure 1). Because the empty IVD was no longer stretched, the disc height was manually kept constant during the injection to avoid an underestimation of the cement volume. The stretch was released once the cement started hardening, to avoid cement leakage through the annulus incision. CT imaging and cement geometry visualization.

**FIGURE 2:**
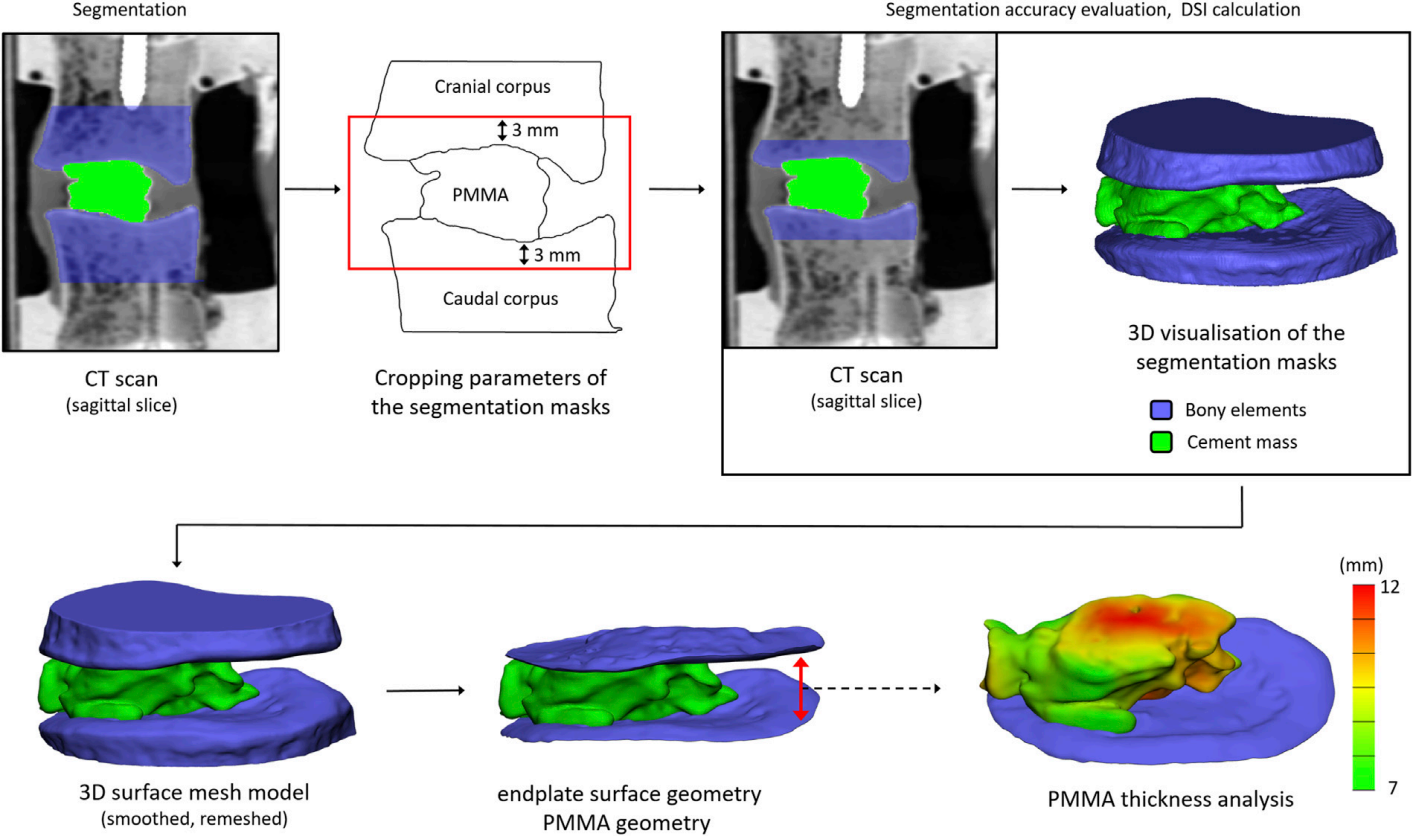
Workflow for the detection of the bony endplates and cement mass to visualize and assess the distribution of the cement in the intervertebral space, and to measure the cement thickness.

In order to study the cement distribution inside of the disc, the specimens were scanned after PCD with a clinical computed tomography scanner (Aquilion ONE, Toshiba) with 220 mA, 120 kV, 0.3 mm slice thickness, 0.214 mm pixel size. The vertebrae and the cement mass were segmented with an image analysis software (Mimics Innovation Suite-v23.0, Materialise, Leuven, Belgium) on the CT slices using thresholding algorithm. Because of the extremity pots used for mechanical testing, the *in vitro* vertebrae were uniformly cropped at 3 mm from the deepest part of the endplate curvature to achieve a region of interest common to all specimens. (Figure 2). All segmentations were performed by two independent operators (C.T and F.B). Segmentation repeatability was measured with Dice Similarity Index (DSI) (Eltes et al., 2020).

The segmented masks were automatically converted into 3D surface meshes, and smoothed (iterations: 6, smooth factor: 0.7, with shrinkage compensation). The geometries were imported and measured (3-Matic 14.0, Mimics Innovation Suite v23.0). The vertebrae and bone cement geometries were first uniformly re-meshed (target triangle edge length: 0.3 mm, surface contour preservation, bad edges removing, split edge factor: 0.2). The endplate surfaces were manually selected. The cement thickness was defined between the two-endplate surface planes, and was measured with the Midplane Thickness Analysis module of 3-Matic.

### 2.5 Biomechanical testing

**FIGURE 3:**
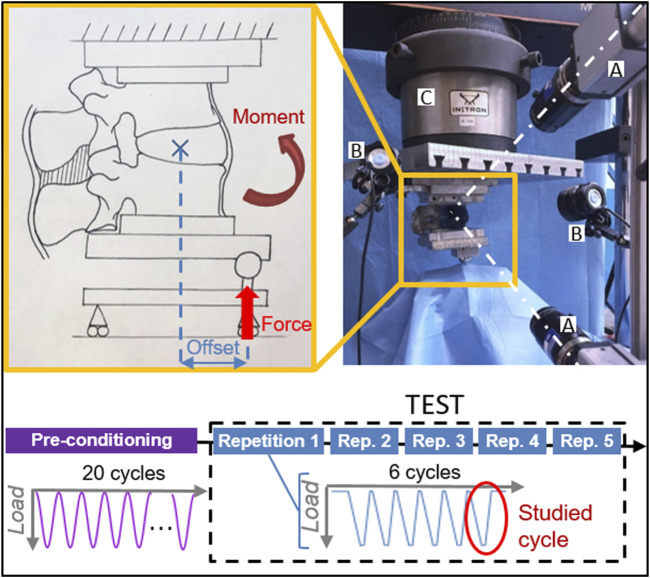
Testing protocol with the experimental setup of the test in flexion and composition of the test sequence. Two cameras (A) targeted the specimen, which was illuminated by high-intensity LEDs (B). The force applied by the testing machine (C) was delivered with an offset, resulting in a combination of a force and a moment.

The scope of our work was to test if discoplasty can provide relief by increasing the foramen space with respect to the degenerated conditions. *In vivo*, one of the most concerning loading scenarios for nerve compression within the foramen is related to a combination of an axial load and motions in a sagittal plane. For this reason, the specimens were mechanically tested in flexion and extension using a uniaxial testing machine (Mod. 8032, Instron, United Kingdom). For these motions, spinal specimens are usually tested under pure moments, even if it was found less physiological for damaged or treated segments in comparison with axial loadings (Wilke et al., 2001). Thus, one pot was rigidly fixed to the top of the testing machine. In order not to constrain the relative motion of the two vertebrae and avoid buckling, the caudal vertebra was loaded through a spherical joint moving along a low-friction rail (Figure 3). This set-up allowed to reach the full load in a relatively fast loading, comparable to the speed one can expect in living subjects (file wp4_130109_1_17 from database OrthoLoad (Bergmann, 2008)).

For each specimen, a force of 50% of the respective Body Weight (BW) of the respective donor, representing the upper body above the lumbar vertebra, was applied with an anterior (posterior) offset, generating a combination of compression and flexion (extension) (Table 1). To have an anatomical definition of the offsets, the lever arms were measured with respect to the centre of the disc on CT images. As the segments are more flexible in flexion, the assigned offset was smaller (35% of the antero-posterior length of the disc) compared to extension (70% of the length) to ensure a similar bending moment was applied in both directions. A reduced load, as close as possible to 50% BW, was applied to some specimens which exhibited a large mobility after nucleotomy, to prevent the endplates from coming in contact and possibly being damaged under the initially planned load (Table 1). Because the loading conditions integrated the body anatomy, the resulting moment would vary between specimens, with a standard deviation of 1.15 Nm in flexion and 2.31 Nm in extension.

**TABLE 1:** Donors’ data and testing parameters for flexion and extension.

Specimen	Sex-age	Lumbar level	Offset (mm)		Axial displacement variation (mm)		Testing load (N)	
			Flexion	Extension	Flexion	Extension	Flexion	Extension
P01	M-68	T12-L1	12.3	24.5	−0.59	−1.35	402	
		L4-L5	14.6	29.3	−0.49	−0.35	402	
P02	M-79	L2-L3	15.1	30.1	–	−0.61	–	387
P03	M-53	L2-L3	13.1	26.3	−1.40	−0.73	402	
		L4-L5	13.6	27.2	−2.21	−0.15	402	
P04	F-35	T12-L1	10.4	20.8	−0.37	−0.21	309	
		L2-L3	10.7	21.5	−3.20	−0.43	309	
		L4-L5	11.0	22.1	–	−0.34	–	309
P05	F-68	T12-L1	9.1	18.2	−2.06	−0.84	396*	
P06	M-59	L2-L3	10.9	21.8	0.49	−0.02	326*	
		L4-L5	11.6	23.2	0.59	−0.52	140*	326*
P07	F-78	L1-L2	12.9	25.8	−0.46	−0.09	348	
		L3-L4	13.4	26.9	1.05	−0.69	348	
P08	M-79	L1-L2	12.8	25.7	−2.02	−1.34	456	
		L3-L4	14.8	29.5	−1.22	−0.68	456	
P09	F-86	L1-L2	13.6	27.2	−1.17	−0.56	265*	
		L3-L4	15.8	31.5	−0.92	−0.61	265*	
P10	M-71	L1-L2	11.9	23.7	−1.98	−0.38	343	
		L3-L4	13.3	26.5	–	−0.40	–	343
P11	M-68	L2-L3	12.6	25.3	−0.44	−0.11	319	
		L4-L5	13.2	26.3	−1.51	−0.01	319	
P12	F-80	L3-L4	13.9	27.9	−0.63	−0.42	378	
P13	M-64	L1-L2	12.8	25.6	−1.62	−0.09	417	
		L4-L5	14.9	29.8	−4.31	0.09	417	
P14	M-73	L3-L4	16.6	33.1	−2.12	−0.57	515	
P15	F-74	L1-L2	12.3	24.6	−1.65	−1.01	412	
		L4-L5	15.4	30.7	−1.16	0.24	412	

*Reduced load to avoid damages.

The loading ramp lasted 1.0 s; the maximum loading was held for 0.3 s, then the specimen was unloaded. Each test consisted of 6 loading cycles, where the last one was analysed in detail (Figure 3). Three cycles are sufficient for minimizing the effect of the viscous component in the response in another study (Cottrell et al., 2006), the subsequent cycles being nearly identical in term of loads and displacements. Each 6-cycle test was repeated five times to assess the repeatability. Before being tested, each specimen was pre-conditioned applying the test load as a sinusoid at 0.5 Hz for 20 cycles. The specimens were tested in nucleotomy and cement discoplasty conditions for both directions of loading. The applied load and the actuator displacement were recorded by an independent datalogger (PXI, Labview, National Instruments, Austin Texas, US) at 500 Hz.

During each test, the 3-dimensional displacements and strain distribution of the specimen surface were tracked using a Digital Image Correlation (DIC) system. This technique requires a high-contrast speckle pattern on both the vertebrae and the intervertebral disc (Figure 1). First, the segment was stained with a methylene blue solution to create a dark background without impacting the properties of the tissues (Lionello, et al., 2014). The white pattern was then sprayed with a water-based acrylic paint, following a procedure optimized elsewhere (Lionello, et al., 2014; Palanca, et al., 2015). Four white dots were manually added along the endplates to accurately identify the disc cranial and caudal borders from the images. To measure the displacements and the deformations over the specimen surface, a 3D-DIC system (Q400, Dantec Dynamics, Skovlunde, Denmark) was optimized (Palanca, et al., 2015) (Table 2) and used (Figure 3). Image acquisition was performed in lateral view with the cameras pointing to the neuroforamen. Images were recorded at 15 Hz from the unloaded condition (reference frame, no load applied) to the end of the sixth cycle. In order to synchronize the DIC images with the testing machine data, the axial translation of the mobile vertebra, corresponding to the actuator motion, was derived from the images. The PXI load-displacement and axial translation curves were then temporally aligned by automatically identifying the peaks and valleys of the cycles.

**TABLE 2:** Material and parameters of the DIC system.

Material	Acquisition	Images post-processing
2 cameras: 5 megapixels, 2440 × 2050 pixels, 8-bit. 26° between the cameras	Software: Instra 4D, v.4.3.1, dantec dynamics	Facet size: 35 pixels
35 mm lenses: Apo-Xenoplan 1.8/35, Schneider-Kreuznach, Bad-Kreuznach, Germany	Calibration: Al4-BMB-9 × 9, dantec dynamics	Grid spacing: 11 pixels
Lights: cold-light LEDs	Field of view: 60 mm × 100 mm Pixel size: 0.04 mm	Filtering: local 7 × 7 pixels kernel

### 2.6 Data analysis

All measurements were compared for each specimen and each loading configuration (flexion, extension) between the two conditions: nucleotomy (NUCL), and percutaneous cement discoplasty (PCD). Since PCD aims to assess the changes of the height of neuroforamen, the posterior disc height (PDH) was measured at the peak load as the cranial-caudal distance between endplates close to the neuroforamen (Supplementary Material S1 File).

The motions (translations and rotations) of each vertebra were computed from DIC images (Morosato, et al., 2019; Techens et al., 2020). The range of motion (ROM) was defined as the relative angle between the vertebrae in the sagittal plane between the peak load and unloaded conditions. Laxity (LZ) and elastic zones (EZ) were identified from load-displacement curves (Supplementary Material S1 File) as respectively the region of large mobility and no loading, and the region where tissue stretched. The transition point defined the limit between the two zones and the elastic stiffness was evaluated on the EZ (M. L. Tanaka et al., 2011).

The maximum and minimum true principal strains (Ɛ1, Ɛ2) over the vertebrae and IVD were measured at the peak load. Their median over the disc surface were computed, as well as their extreme values (defined as the 95%-percentile, to avoid local measurement artifacts).

All the computations were performed with dedicated Matlab scripts (MathWorks Inc., Natick, MA, United States). To overcome the inter-specimen variability, the parameters measured after cement discoplasty were normalized to the nucleotomy condition of the respective specimen.

### 2.7 Statistical analysis

Shapiro-Wilk tests were applied to all parameter distributions to assess their normality (α = 0.05). Depending on the normality assessment, comparisons between nucleotomy and discoplasty were made for ROM, stiffness, height, and the strain median with either a non-parametric Wilcoxon’s test or a paired *t*-test. Influence of the spine level on the results was assessed with a one-way ANOVA. Finally, correlations between the cement distribution and the biomechanical parameters were evaluated with Spearman’s rank correlation coefficient using SPSS Statistics 25.0 (IBM Corp., Armonk, NY, United States) with *p* = 0.05. The interpretation of the correlation strength was based on Evans’ classification (Evans, 1996) (ρ < 0.20 is very weak, 0.20 to 0.39 is weak, 0.40 to 0.59 is moderate, 0.60 to 0.79 is strong and 0.80 or greater is a very strong correlation).

## 3 Results

The different indicators were normalized between NUCL and simulated PCD for each specimen and each direction of loading. Main trends are reported here, the detailed parameter values are found in Supplementary Material S1 File.

Two specimens were excluded in flexion: for one, the posterior process broke but the specimen was unaffected in extension; a third specimen broke during the test. One specimen had a DIC-correlated area too small and was only used to measure the stiffness.

### 3.1 Posterior disc height

**FIGURE 4:**
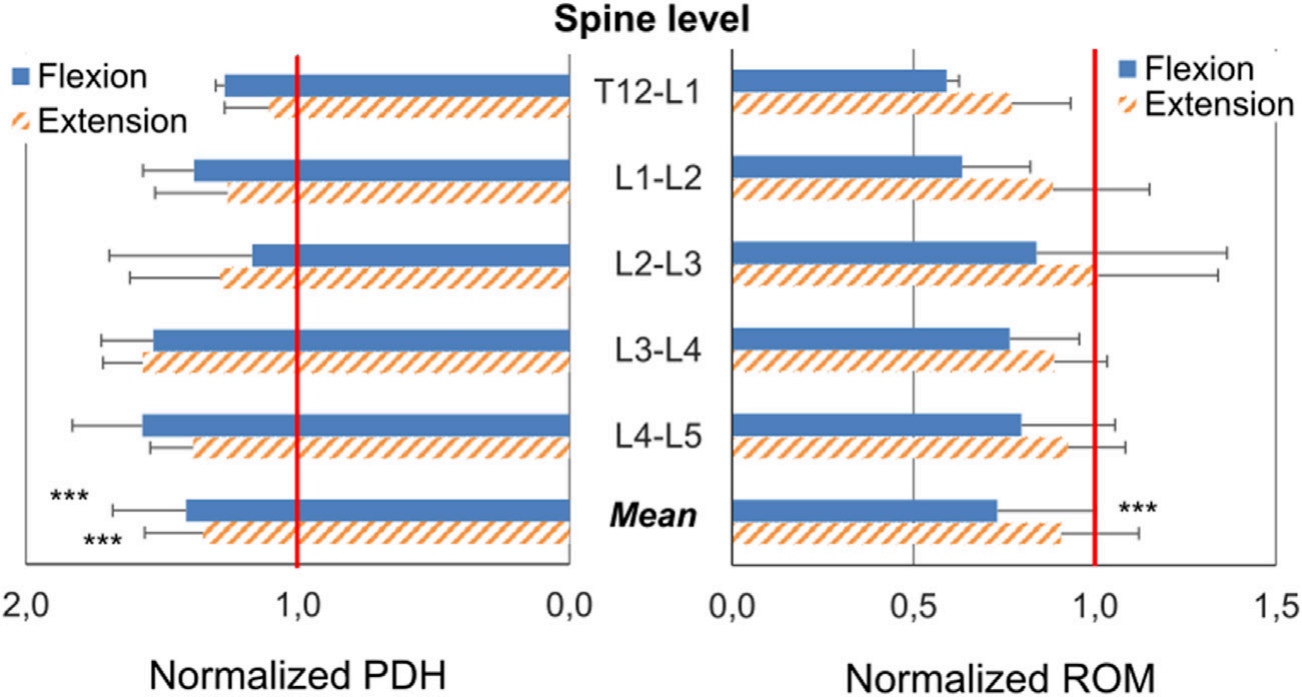
Changes (Mean ± SD) of the posterior disc height (PDH) and of the Range of Motion (ROM) caused by cement discoplasty at the different spine levels and as an average of all levels. The PDH and ROM after discoplasty were normalized with respect to the value before discoplasty: a normalized value of 1.00 indicates no change; a value greater (smaller) than 1.00 indicates that the respective magnitude was increased (decreased) due to discoplasty. First quartile, median and third quartile are represented by lines. Mean is indicated by the cross and min and max values by the whiskers. Statistical significance (paired *t*-test, *p* < 0.001) is designated by ***.

The posterior disc height (PDH) was measured from DIC correlations for flexion (*n* = 24) and extension (*n* = 27). The specimens exhibited a PDH increase of 41% ± 46% (mean ± SD) in flexion (paired *t*-test, *p* < 0.001) and 35% ± 38% in extension (*p* < 0.001). In particular, the largest increase of PDH in both flexion and extension were respectively measured at the L3-L4 level whereas the smallest increase happened at T12-L1 (extension) and L2-L3 levels (flexion) (Figure 4). However, spine level did not significantly impact PDH (ANOVA; *p* = 0.69 in flexion, *p* = 0.65 in extension).

### 3.2 Range of motion

The range of motion (ROM) was derived from DIC correlations in flexion (*n* = 24) and extension (*n* = 27). Discoplasty decreased the ROM by 27% ± 27% (mean ± SD) in flexion (paired *t*-test, *p* < 0.001) and decreased it by 9% ± 96% in extension (*p* = 0.33). The different spine levels exhibited different trends in flexion with a mean ROM drop about 40% for segments between T12 and L2, whereas the low lumbar spine showed a smaller decrease about 20% (ANOVA, *p* = 0.66) (Figure 4). Conversely, similar ROM was measured in extension, independent of the spinal segment (*p* = 0.56).

### 3.3 Stiffness

**FIGURE 5:**
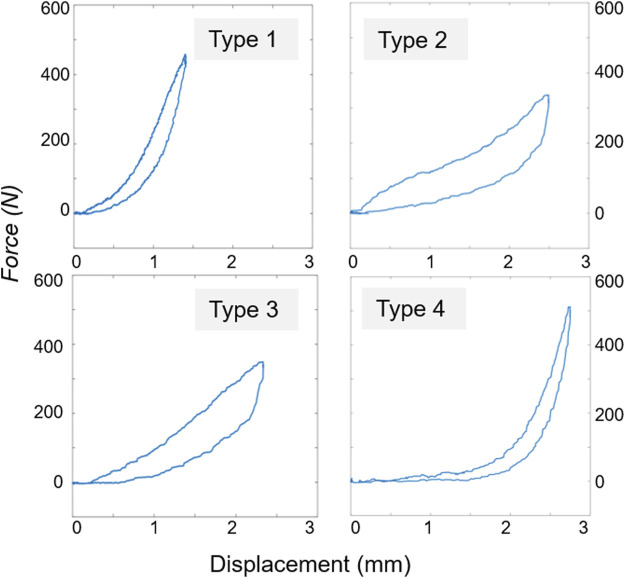
Typical load-displacement curves found for the 27 specimens depending on the testing conditions (disc condition, motion) resulting in 104 tests. Type 1: the majority of the tests (79/104) was described with an exponential toe region, and linear elastic part which were fitted following Tanaka et al., 2011 model. Type 2: S-shaped was followed by 2/27 specimens in nucleotomy flexion and 7/27 in cement discoplasty flexion. Type 3: 1/27 specimens in nucleotomy extension, 1/27 in cement discoplasty extension, and 4/27 in discoplasty flexion followed a flat toe region and linear elastic region. Type 4: L-shaped was followed by 10/27 specimens in nucleotomy extension.

The specimens showed different behaviours according to the loading configuration and disc condition (Figure 5). After nucleotomy, in flexion load-displacement was described by an exponential curve for the LZ and a linear curve for the EZ in equivalent proportion. In extension, the loading phase showed a flat linear LZ associated with a sharper transition to the linear EZ. After PCD, different behaviours were observed in flexion. Some specimens exhibited an S-shaped load-displacement curve, others a very short LZ followed by a linear EZ. The rest displayed the usual exponential-linear shape already observed after nucleotomy. In extension, specimens followed an exponential-linear behaviour too.

The elastic stiffness, transition load and displacement were estimated by fitting the load-displacement curves (Supplementary Material S1 File: Supplementary Table S1_1, Supplementary Figure S1_2). In flexion, mean transition displacement was reduced by 38% from nucleotomy to cement discoplasty (paired *t*-test, *p* < 0.001) while transition load was unaffected (*p* = 0.28). In extension, both parameters respectively dropped by 28% and 16% (paired *t*-test, *p* < 0.001). For both loading configurations, the LZ was larger after PCD. Finally, discoplasty increased the mean elastic stiffness at peak load by 37% (one sample *t*-test, *p* < 0.01) in flexion but decreased it by 7% (*p* = 0.07) in extension.

When sorting the results by spine level, differences were more pronounced in flexion: the high lumbar spine showed a shorter LZ with very low variability between specimens. Similarly, the variability between specimens was smaller for high levels in flexion. Conversely, in extension, results showed similar trends for all the spine levels.

### 3.4 Strain

**FIGURE 6:**
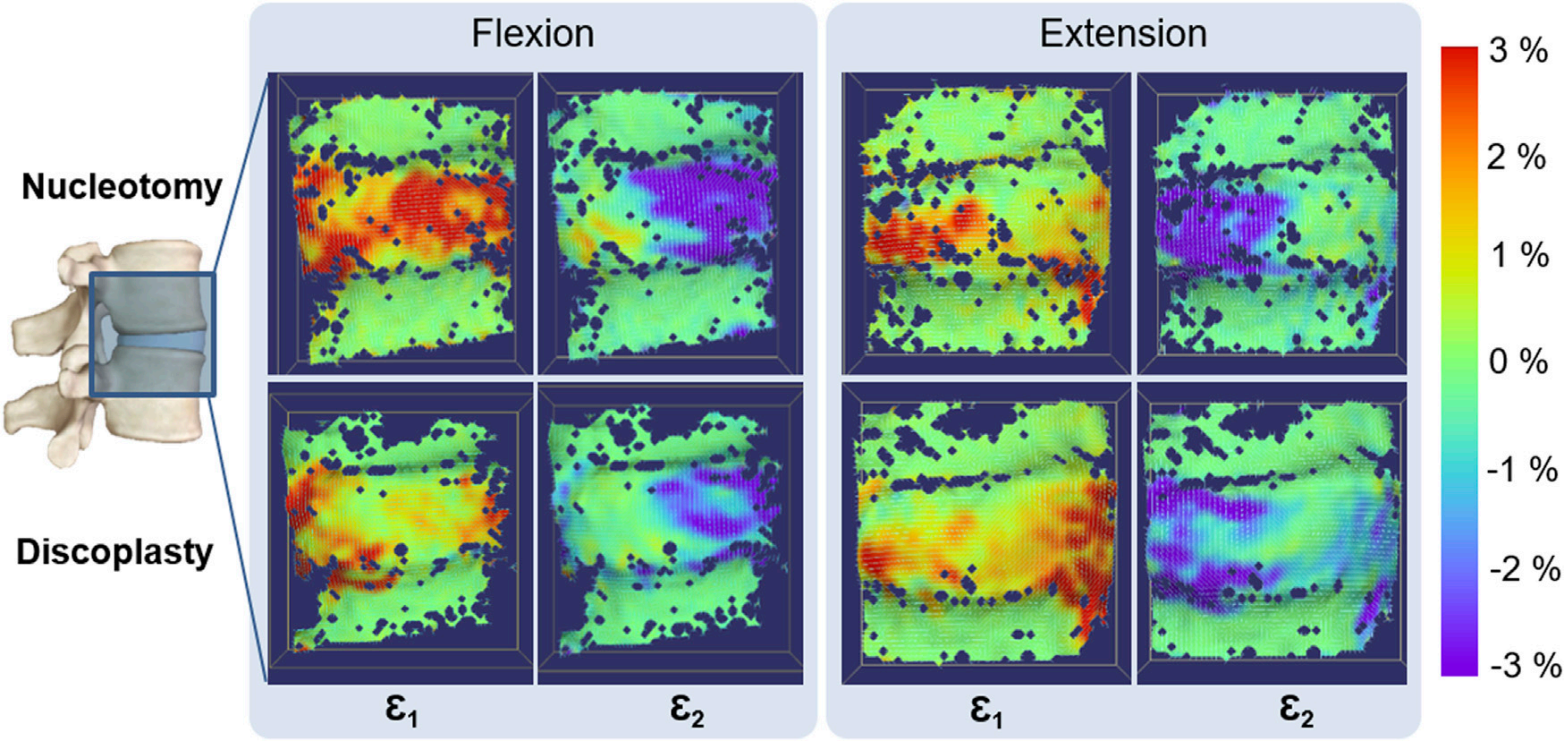
Typical distribution of Maximum (Ɛ_1_) and Minimum (Ɛ_2_) True Principal strains on the specimen surface in flexion and extension. The dark blue spots on the specimen surface correspond to the areas where the DIC algorithm could not correlate, in particular along the endplates. Only the disc underwent large strains.

The mean true strains were derived between specimens (Supplementary Material S1 File: Supplementary Table S1_1, Supplementary Figure S1_3). In flexion, the maximum strain after discoplasty was 12% (paired *t*-test, *p* < 0.05) smaller than after nucleotomy and the minimum strain 40% higher (Wilcoxon test, *p* < 0.01). In extension both strains were reduced by discoplasty by 12% (Wilcoxon test, *p* < 0.05) and 14% (paired *t*-test, *p* < 0.01). These changes of the median value after PCD were associated with a reduction of the extreme strain values, and a shrinkage of the highly strained regions. Discoplasty was associated with a migration of the maximum strains towards the endplates, while minimum strains were located at the disc mid-height (Figure 6). In nucleotomy, in the compressive side of the disc the maximum (tensile) principal strains were directed circumferentially and the minimum (compressive) ones axially. After discoplasty, in the compressive part of the disc the minimum principal strains were directed axially and in the stretched part, the maximum strains were directed circumferential. We also analyzed the number of specimens in which strains exceeded 5%, 10% and 15%. The number of specimens showing high strain values was smaller after PCD (Supplementary Material S1 File: Supplementary Figure S1_4). After discoplasty, the strains did not exceed 10% except in flexion where the number of specimens exhibiting maximum strains exceeding 10% increased. Finally, the most extreme maximum and minimum strains values measured among all specimens were respectively 13.6% and −22.9% regardless the disc condition.

### 3.5 Visualization of the cement geometry and thickness measurement

The inter-operator DSI for the cement and the vertebrae were 0.98 ± 0.02 and 0.97 ± 0.01 (mean ± SD) respectively, indicating a high repeatability. For consistency, the cement distribution over the caudal vertebral endplate in the intervertebral space was visually assessed in the same view for all specimens (Supplementary Material S1 File: Supplementary Figures S1_5,S1_6). In most cases, the cement distributions mimicked the nucleus shape with an average volume of 4.56 ± 1.78 ml (mean ± SD). Surfaces of both endplates and the injected cement were also measured (Table 3). Seven specimens exhibited leakage of cement into at least one vertebral body in different proportions (Supplementary Material S1 File: Supplementary Figure S1_7). The cranial-caudal thickness of the cement was 9.62 ± 1.30 mm (mean ± SD among specimens, Table 3) with larger values for specimens with perforation of the endplate (Supplementary Material S1 File: Supplementary Figure S1_6).

**TABLE 3:** Summary of the cement distribution analysis for each specimen. The cement thickness between the endplates was measured axially.

Specimen	Level	Cement geometry		Cement thickness				Caudal endplate	Cranial endplate
		Surface (cm^2^)	Volume (cm³)	Min (mm)	Max (mm)	Mean (mm)	SD (mm)	Total surface (cm^2^)	Total surface (cm^2^)
P01	T12-L1	18.94	4.18	4.6	9.3	7.4	0.9	15.25	14.98
	L4-L5	21.77	4.12	5.6	13.5	9.5	1.8	13.99	13.98
P02	L2-L3	35.12	7.89	6.5	14.0	10.1	1.6	22.56	21.86
P03	L2-L3	23.72	5.05	6.2	14.3	9.3	1.4	17.82	18.44
	L4-L5	21.67	5.27	6.8	15.0	10.5	1.5	19.90	18.13
P04	T12-L1	11.81	2.14	6.0	10.1	8.4	0.8	12.45	12.08
	L2-L3	16.61	3.40	7.4	12.3	9.6	1.0	13.97	13.79
	L4-L5	13.49	2.02	6.5	12.6	10.5	1.4	13.35	14.00
P05	T12-L1	12.93	2.34	3.4	11.1	6.6	1.3	8.48	9.44
P06	L2-L3	11.49	2.49	5.8	16.9	8.9	1.7	10.89	11.02
	L4-L5	15.35	3.18	4.3	13.8	8.0	1.6	12.14	12.67
P07	L1-L2	21.84	2.86	7.5	19.8	10.8	2.4	21.66	16.58
	L3-L4	20.03	5.07	7.1	16.0	10.6	1.3	16.34	15.56
P08	L1-L2	26.84	7.04	3.8	16.8	9.0	2.3	17.86	18.55
	L3-L4	22.38	3.88	5.3	12.2	9.8	1.6	20.25	19.94
P09	L1-L2	26.12	6.02	4.6	13.8	9.3	1.5	18.81	18.36
	L3-L4	38.70	8.89	5.2	17.2	9.9	1.7	19.72	21.35
P10	L1-L2	24.28	5.51	4.8	15.7	9.9	1.8	15.26	14.77
	L3-L4	24.74	5.44	6.6	18.3	11.6	2.0	16.01	15.98
P11	L2-L3	16.07	3.36	6.4	11.6	9.7	1.3	15.37	15.20
	L4-L5	17.03	3.31	7.1	13.1	10.4	1.3	14.32	14.92
P12	L3-L4	20.14	3.52	2.4	11.9	8.5	2.2	18.12	17.99
P13	L1-L2	22.95	5.69	7.3	15.9	10.9	1.5	15.53	14.89
	L4-L5	27.29	5.15	9.3	16.6	13.2	1.3	16.00	16.05
P14	L3-L4	30.31	7.15	4.3	14.3	9.3	1.8	21.52	20.83
P15	L1-L2	16.51	3.57	5.7	13.9	9.5	1.3	14.25	14.67
	L4-L5	19.40	4.42	3.7	14.8	8.8	2.2	18.25	18.67
	**Mean**	**21.39**	**4.56**			**9.6**		**16.30**	**16.10**
	**(SD)**	**(6.65)**	**(1.78)**			**(1.3)**		**(3.44)**	**(3.10)**

The bold values are indicated to be Mean and Sd.

### 3.6 Correlation between cement geometry and biomechanical parameters

**FIGURE 7:**
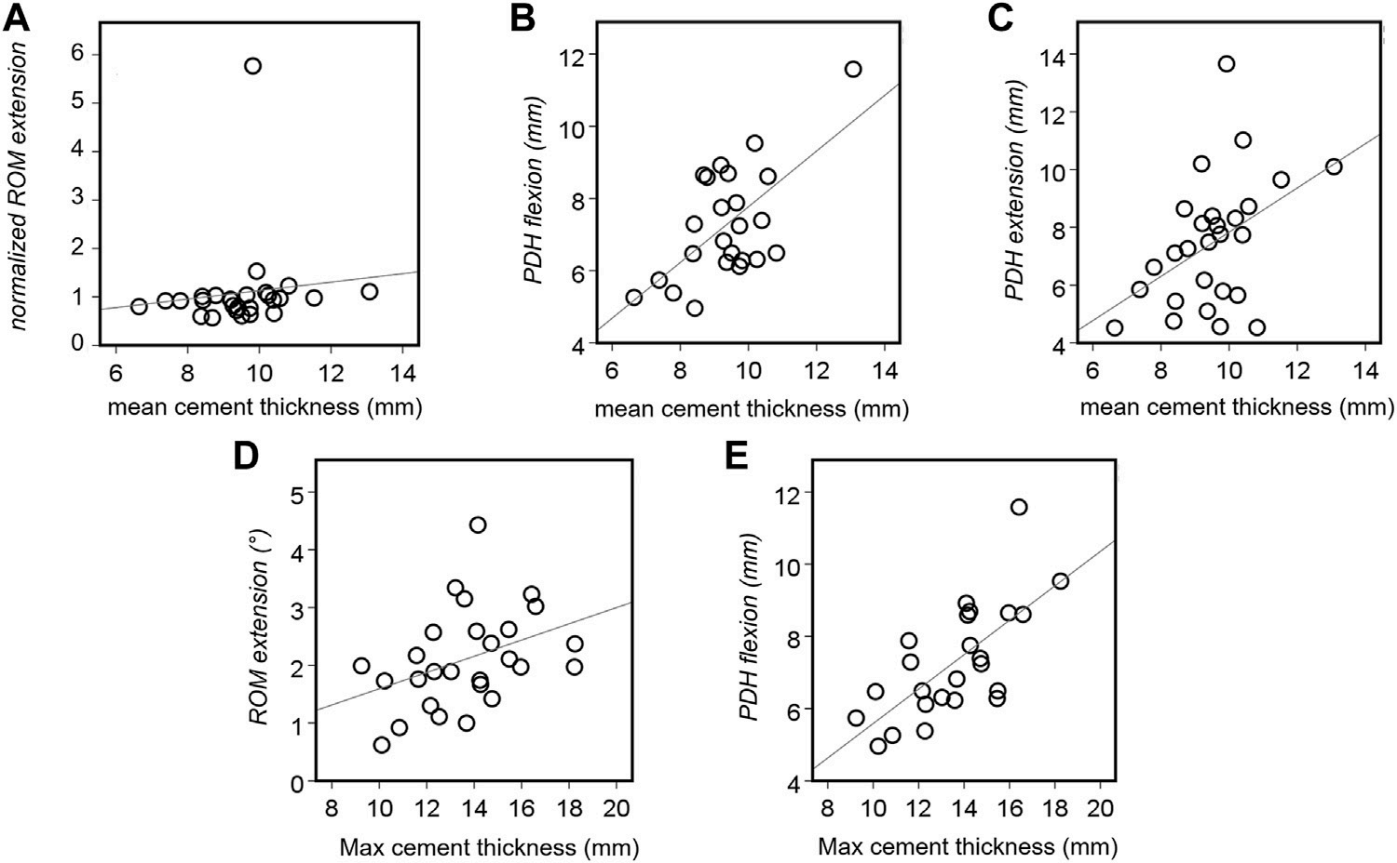
Statistically significant associations between the cement thickness and the biomechanical parameters of the tested specimens. Correlations were found for the ROM in extension (**(A)**: *p* = 0.037 and **(D)**: *p* = 0.032), the PDH in extension (**(B)**: *p* = 0.048) and flexion (**(C)**: *p* = 0.034 and **(E)**: *p* < 0.001).

Relationships between cement distribution and biomechanical parameters were investigated for each direction of loading (Figure 7). The mean cement thickness positively and moderately correlated with PDH after discoplasty in both extension and flexion (Spearman’s coefficient *ρ* = 0.410, *p* = 0.034, C and *ρ* = 0.407, *p* = 0.048, B). It also moderately affected the normalized ROM in extension (*ρ* = 0.432, *p* = 0.037, A). Similarly, the maximum cement thickness strongly affected PDH in flexion (*ρ* = 0.663, *p* < 0.001, E) and showed a moderate positive correlation with the absolute ROM in extension (*ρ* = 0.413, *p* = 0.032, D). Relations with other parameters were not significant.

## 4 Discussion

This study assessed the biomechanical consequences of PCD on the spine kinematics and on the strain distribution. To investigate the effects of PCD on the intervertebral range of motion and stiffness, and on the strain distribution, 27 FSUs were prepared with a simulated disc lesion, and then treated with cement discoplasty. Simulated discoplasty was found to significantly increase the intervertebral height in the posterior region both when flexion and extension were applied.

Discoplasty also impacted the segment kinematics, significantly reducing the flexibility in flexion. This was associated with a shortening of the laxity zone and an increase of the elastic stiffness. The difference of load-displacement behaviours after nucleotomy and after discoplasty were caused by the combination of the action of the elements of the intervertebral joint. In flexion, facet capsules and posterior ligaments were shown to transmit the load (Tencer, et al., 1982). Different from nucleotomy condition, where the vertebrae are free to rotate until the posterior elements stretch (exponential + linear behaviour), the presence of cement already spaced the facets and pre-stretched the posterior ligaments. This resulted in an immediate loading of the facet capsules followed by the posterior ligaments explaining the two load increases of the S-shape.

Conversely, the cement mass did not affect the mobility in extension. It only impacted the beginning of the segment motion, shortening the laxity zone: during extension, the motion of the vertebrae, “rolling” on the cement, is only constrained by the contact of the posterior elements independently to the presence of cement. Discoplasty also modified the load-displacement behaviour. Following nucleotomy, the intervertebral joint only transmitted the load after the facets contact, resulting on a suddenly stiffening behaviour (L-shape). Discoplasty smoothed this behaviour, probably restoring part of the role of the joint elements. A study suggested that anterior longitudinal ligament had a limited effect on load-resistance in extension attributing it to the bulk compression in the posterior of disc (Tencer, et al., 1982 Ahmed and Burke, 1982).

Discoplasty also reduced the disc tissue deformation: in average, both the maximum and the minimum strain were lower than after nucleotomy. After nucleotomy a large bulging was induced in the disc under load in particular on the compressed side of the disc, leading to both high tensile and compressive strains in the same location at mid-height. Conversely, once the disc height was restored by PCD, the anatomical elements retrieved their functions in the spine motion, with tensile strains located on the ALL in extension and in the posterior disc in flexion. The distribution of compressive strains became more defined after PCD, with concentrations along the endplates. The largest values of strain found on the surface were reduced after discoplasty. Comparing the corresponding stretches, discoplasty induced values within the same range as estimated strains *in vivo* (Long et al., 2016). Thus, discoplasty did not seem to present a risk of damage for AF outer tissues.

The injected cement was largely distributed in the disc space. In some cases, the injection process resulted in perforation of the endplates, due to degenerative lesions or deterioration caused by the nucleotomy. Cement perforation of the endplates is a clinically known phenomenon which does not represent a contraindication to PCD (only leakages into the spinal canal are represent a relevant clinical complication, and must be treated accordingly (Varga et al., 2015; Sola et al., 2018; Willhuber et al., 2019)). Cement geometry directly impacted PDH and ROM. The thicker the cement, the higher PDH for both directions of loading. The thickness also induced a significant positive moderate correlation with the ROM in extension as a consequence of a larger PDH. Indeed, extension motion is restricted by the contact of the facets. A high PDH spaced the cranial and caudal facets giving a wider range of mobility in extension.

As presented in clinical studies, PCD aims to recovering the healthy disc height and neuroforamen section by creating a cement spacer between the vertebral bodies. This study supported clinical observations (Varga et al., 2015; Sola et al., 2018; Kiss et al., 2019), presenting a significant increase of PDH under bending, which is a more critical loading scenario with a reduced foramen than in *in vivo* measurements made in supine/prone position. In addition, a clear reduction of the stress in the nerve root after surgery was numerically reported compared to the degenerated case (Jia, et al., 2022).

Percutaneous cement discoplasty application to lumbar spine is a recent surgical technique. The only paper about *in vitro* biomechanical testing which can be found in literature was performed on porcine lumbar discs (Techens et al., 2020). Different from the present human study, that preliminary porcine study did not report any significant change of ROM nor stiffness after PCD, but reported similar changes in the strain distribution concluding that discoplasty procedure tends to restore the deformation state of a healthy disc. The difference between the present findings and the previous study probably relates to the difference between human and porcine in terms of NP and AF, and anatomy of the facets. The ROMs measured at peak load were in the same range as others *in vitro* studies on human spines (Markolf, 1972; Posner et al., 1982; Janevic, et al., 1991; Spenciner et al., 2006; Heuer et al., 2007; Heuer, et al., 2008; Zirbel et al., 2013; Amin et al., 2016). Other studies on the effect of nucleotomy demonstrated that the absence of NP reduced segmental rotational stability, significantly increasing the ROM (Johannessen et al., 2006; Wilke et al., 2006; Heuer et al., 2007; Russo et al., 2017). Only Eysel *et al.* found a drop of ROM for both motions (Eysel et al., 1999). Heuer *et al.* presented the strain map of intact IVDs, exhibiting similar distributions with the strain measured in this present study after PCD (Heuer, et al., 2008). Experimental results could also be put in perspective with *in silico* study findings. Li *et al.* showed that PCD reduced the maximum stresses in the annulus tissue for both flexion and extension in comparison to intact disc (Li et al., 2022), supporting the conclusion drawn here from the strain map. They also reported an increase of stress in the endplates for both directions of motion, in particular below the disc. Such asymmetry did not clearly appear in the experimental strain distribution, neither in another numerical study (Jia, et al., 2022).

In parallel, clinical studies investigated the surgical procedure. The *in vivo* cement masses presented by Eltes *et al.* (Eltes et al., 2021) almost filled the disc volume. In our case, donors were relatively old, but still had a very strong annular structure. Removing it to only have the outer layer like in advanced degeneration with vacuum was very difficult considering that the nucleus was removed by a spine surgeon using standard surgical tools. Then, the cement volumes injected in our study entirely fit the nucleus space with values close to the range of 3–5 ml clinically reported (Varga et al., 2015). Finally, one should highlight the lack of bone cement extrusion through the AF defect at the end of testing. Indeed, extrusion of the filling material is a major concern in the research of NP regeneration techniques, particularly when the AF is damaged to allow the material insertion (Wilke et al., 2006). Therefore, PCD does not require AF repair.

One limitation relates to the simplified loading conditions applied. FSUs *in vitro* are usually subject to pure bending moments (Wilke, et al., 1998) sometimes coupled with a compressive preload (Janevic, et al., 1991; Gardner-Morse and Stokes, 2003; Zirbel et al., 2013). The setup used in this study applied an eccentric compressive load which induced the bending, as in other spine studies (Adams and Dolan, 1996; Flamme et al., 2006; McNally, et al., 2012). Although the pivot point does not remain stationary during the motion, the lever arm variation during the tests was evaluated by DIC in term of relative translation of the vertebra. This resulted in a change of the bending moment between nucleotomy and discoplasty conditions of 3.2% ± 2.8% (mean ± SD) in flexion and 1.1% ± 1.0% in extension, making the loading conditions comparable.

Due to the diverse degeneration states of the donors’ intervertebral discs and the limited number of available specimens, nucleotomy was required to establish a common condition allowing possible repetition of the study. For that, fenestration in the AF was needed, which can be suspected to compromise the biomechanics of the FSU. The impact of fenestration on the segment stability was assessed in a dedicated experiment (Supplementary Material S2 File). The PDH, ROM, stiffness and strain distribution were checked. Fenestration of the AF did not significantly perturb the disc behaviour in comparison to the nucleus extraction/removal. Nucleotomy only significantly reduced the disc height. Lateral bending was not investigated because the position of the defect would have prevented the test of both bending directions.

## 5 Conclusion

This study was the first to investigate the impact of cement discoplasty on the biomechanics and kinematics using *in vitro* human lumbar spines. Because *in vitro* testing only tries to model the *in vivo* conditions, the clinical integration of the result absolute values is limited. However, general assumptions can be drawn from the comparative data and integrated into clinics.

• The cement filled the empty discs and *in vitro* distributions had similar volume and thickness as clinically observed.
• The posterior disc height was increased after discoplasty with respect to the nucleotomy condition: the cement mass acted like a spacer, supporting clinical observations.
• Stability of the segment was greater in flexion after discoplasty: the range of motion was significantly reduced, and the elastic stiffness increased.
• No indication of risks of mechanical damage on the outer disc was observed after discoplasty: the distribution of strain on the disc showed a clear decrease of large disc deformations.
• The cement geometry, in particular cement thickness, directly influenced the posterior disc height, and impacted the range of motion in extension only.

## Data Availability

The datasets generated for this study can be found in the Figshare repository “Biomechanical consequences of cement discoplasty: An *in vitro* study on thoraco-lumbar human spines” https://doi.org/10.6084/m9.figshare.19145255, https://doi.org/10.6084/m9.figshare.19145411, https://doi.org/10.6084/m9.figshare.19145075, https://doi.org/10.6084/m9.figshare.19145213, https://doi.org/10.6084/m9.figshare.19145231, https://doi.org/10.6084/m9.figshare.19145372, https://doi.org/10.6084/m9.figshare.19145363, https://doi.org/10.6084/m9.figshare.19145348, https://doi.org/10.6084/m9.figshare.19145378.
