# Supplementary material for: Biomechanical consequences of cement discoplasty: An in vitro study on thoraco-lumbar human spines
Source: Front Bioeng Biotechnol. 2022 Dec 2;10:1040695. doi: 10.3389/fbioe.2022.1040695 (PMC9755512; doi:10.3389/fbioe.2022.1040695)
Supplement: Supplementary file 1 [file DataSheet2.PDF]

Supplementary Material #2

The effect of intervertebral disc simulated damage

Appendix to the paper

Biomechanical consequences of cement discoplasty: an *in vitro* study on thoraco-lumbar human spines

1 Introduction

In vitro biomechanical investigation of percutaneous cement discoplasty (PCD) requires collateral lesions, such as annulus incision and disc material removal, to recreate the typical vacuum phenomenon of the degenerated discs that would require PCD. The mechanical impact and consequences of annulus lesions on spine biomechanics have not been completely assessed (Galbusera et al., 2014; Kirkaldy-Willis and Farfan, 1982; Tanaka et al., 2001; Thompson et al., 2004, 2000).

The aim of this set of experiments was to evaluate the consequences of intervertebral disc lesions on the spine biomechanics, especially due to the incision of annulus fibrosus as those performed in the main paper, in order to:

i. Explore the artefacts induced by simulated disc lesions, in terms of disc height, stiffness and range of motion.

ii. Assess to what extent simulated nucleotomy affects the biomechanics of the IVD.

More specifically, this additional study aimed to assess if the lesion of the annulus required to remove the nucleus would compromise the biomechanics of the FSU significantly, compared to the alteration caused by the simulated disc degeneration.

2 Materials and Methods

2.1 Specimens, imaging, and preparation

Eight fresh cadaver thoraco-lumbar functional spine unit were extracted from six donors (Table S2_1). CT scans were performed for all the spines, (Aquilion, ONE, Toshiba, Bologna) in the same conditions of the main paper.

The FSUs were prepared following the same protocol as the main paper, except for the posterior processes, which were cut at the facet joints level. In addition, osteophytes were removed in specimens #1 and #2, ensuring not to damage the disc.

Table S2_1 - Donors' data with testing parameters for flexion and extension.

Specimen N°	*Level*	*Sex – Age*	*Offset (mm)*		*Applied Load (N)*	*Width of the slot (mm)*
			Flexion	*Extension*		
#1	T9 - T10	F - 77	8.5	17.0	211	4.2
#2	T11 - T12	F - 77	9.3	18.6	211	6.2
#3	T10 - T11	M - 68	10.5	21.0	402	5.3
#4	L4 - L5	M - 79	17.3	34.6	388	10.5
#5	T12 - L1	M - 53	11.7	23.4	402	5.4
#6	T10 - T11	M - 59	13.1	26.2	495	5.6
#7	T12 - L1	M - 59	13.4	26.8	495	5.9
#8	T10 - T11	F - 58	8.2	16.4	*300*	5.9
Mean		66	11.5	23.0	363	6.1
SD		10.3	3.1	6.1	112.6	1.9
Range		53 - 79	8.2 – 17.3	16.4 – 34.6	211 - 495	4.2 – 10.5

2.2 Incremental creation of the defects in the disc

In order to explore how the incisions on the annulus affect the biomechanics of the FSU, all the specimens were sequentially tested, through these five subsequent conditions (Fig. S2_1):

a) INT: the disc was in the native conditions (intact)
b) 2CUTS: two vertical cuts from the upper endplate to the lower endplate were made on the lateral side of the annulus fibrosus as deep as required to reach the nucleus. The two incisions were spaced by a mean of 6.1 mm (range 4.2 – 10.5 mm)
c) 4CUTS: two additional horizontal cuts were made along the endplates, connecting the first two cuts so as to form a square incision on the annulus tissue.
d) SQR: the square-shaped plug of annulus tissue was removed, leaving the nucleus visible.
e) NUCL: the nucleus pulposus was extracted with curette and surgical spoon through the window created in the previous condition. This final condition corresponded to the starting condition (NUCL) of the specimens tested for discoplasty in the main paper.

In order to minimize variability between testing session, the lesions 2CUTS, 4CUTS and SQR were created while the specimen was on the testing machine. Removing all the nucleus pulposus from the FSU on the testing machine was not possible. Therefore, the nucleotomy and the subsequent mechanical tests were performed at a later time.

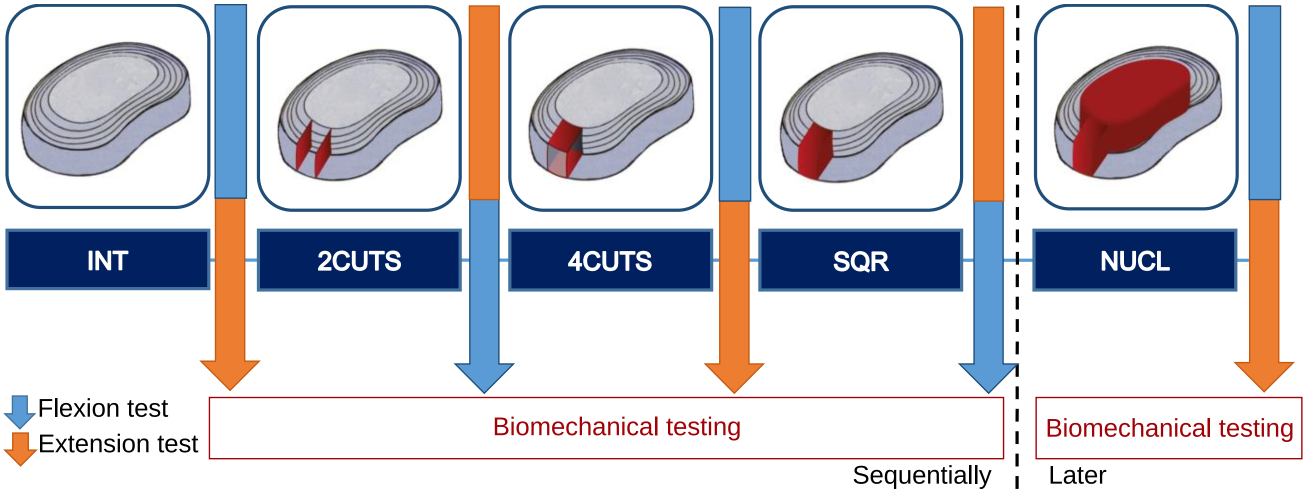

Fig. S2_1 – Different steps of treatment of the IVD (the red parts represent the cuts and the removed materials) and block diagram of the testing protocol: all the tests until SQR were performed at the same session, NUCL tests were performed later.

2.3 Mechanical testing and measurement of displacements and strains with DIC

The tests were sequentially performed, and the disc defect was increased after testing in both loading configurations (Fig. S2_1). The specimens were mechanically tested with the same protocol described in the main paper and undergoing the same loading configurations (Fig. S1_1 in the Online Resource). For specimen #8, a load lower than the standard 50% BW was applied to avoid specimen damage. Axial load and displacement were acquired as in the main paper.

The displacements and strains were measured using Digital Image Correlation (DIC). The same hardware and parameters as in the main paper were used, the only difference being a slightly different grid spacing, which was set at 19 pixels to minimize the errors over the selected field of view.

3 Results

3.1 Posterior disc height

Posterior disc height was measured on DIC correlated images (Fig. S2_2). Normalized data showed a monotonic decrease from INT to NUCL both in flexion (-20% height) and extension (-23% height) (Table S2_2). The overall trend was statistically significant (Friedman test, $p=0.036$ in flexion and $p=0.016$ in extension). Relative comparison between the different disc conditions indicated that the only significant differences were between 4CUTS and NUCL in extension (Nemenyi test, $p = 0.019$). Large differences were observed also between nucleotomy and 2CUTS and 4CUTS in flexion and between nucleotomy and 2CUTS in extension, but they were poorly significant (Nemenyi test, $p=0.057$).

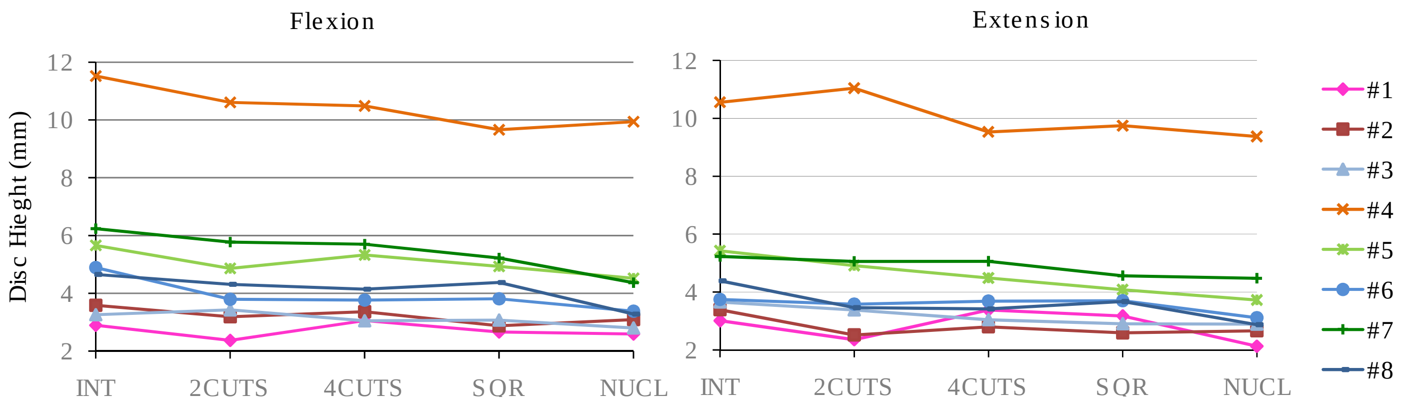

Fig. S2_2- Disc height measured in the posterior region (PDH) in the different conditions, starting from intact (INT), and with progressive steps of incision (2CUTS, 4CUTS, SQR) in the annulus fibrosus, and after nucleotomy (NUCL). The mean values over three measurements for each of the eight specimens are plotted for flexion and extension.

Table S2_2 – Posterior disc height (PDH) in flexion and extension for the five conditions, normalized for each specimen with respect to the respective intact condition. The mean of eight specimens is reported. A value smaller than 1.00 indicates a decrease of the disc height with respect to the intact.

	Posterior Disc Height	
	Flexion	*Extension*
INT	1.00	1.00
2CUTS	0.90	0.89
4CUTS	0.92	0.91(*)
SQR	0.87	0.87
NUCL	0.80	0.77

Note: The asterisks indicate where a statistically significant difference was found with respect to the intact (Nemenyi test) (*) $p<0.05$; (**) $p<0.01$; (***) $p<0.001$.

3.2 Range of Motion

From the measured three-dimensional motions, the component in the sagittal plane was extracted (Table S2_3). There was no statistically significant difference between the five conditions, both in flexion (Friedman test, p= 0.20) and in extension (Friedman test, p= 0.48). Despite this, a general increase of the ROM due to the 2CUTS, and a decrease after nucleotomy were observed in flexion (Fig S2_3) and a generally decreasing trend of the ROM was observed in extension.

Table S2_3 – Range of motion (ROM) in flexion and extension for the five disc conditions, normalized for each specimen with respect to the respective intact conditions. The mean of eight specimens is reported. A value larger than 1.00 indicates an increased ROM with respect to the intact condition.

	Range of motion	
	Flexion	*Extension*
INT	1.00	1.00
2CUTS	1.09	0.98
4CUTS	1.03	1.16
SQR	1.04	1.06
NUCL	0.94	1.22

Note: The asterisks indicate where a statistically significant difference was found with respect to the intact (Nemenyi test) (*) p<0.05; (**) p<0.01; (***) p<0.001.

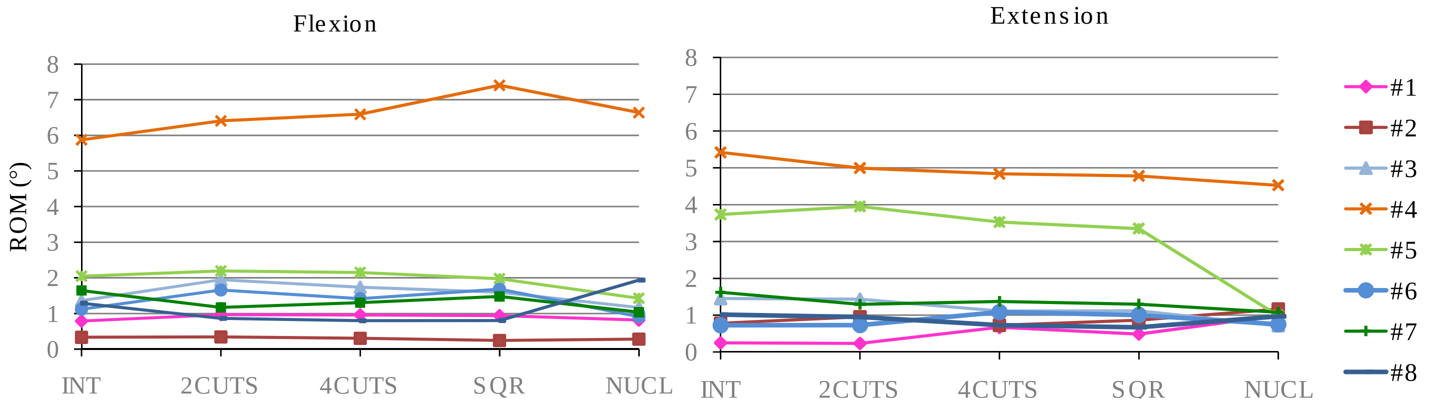

Fig. S2_3 – Range of Motion (ROM) in the different conditions, starting from intact (INT), and with progressive steps of incision (2CUTS, 4CUTS, SQR) in the annulus fibrosus, and after nucleotomy (NUCL). The median values over the five test repetitions are plotted for flexion and extension.

3.3 Stiffness

The load – displacement curves showed different trends between specimens and for each type of motion. In flexion, the load – displacement curves showed monotone or exponential shape for all specimens. An increase of the sigmoid-shape curve in the laxity zone was common to the specimens: usually the first part of the curve was linear. In extension, the specimens showed a linear behaviour except one specimen with an exponential one. Both in flexion and in extension, nucleotomy condition exhibited a comparable or lower slope respect to the others.

The transition displacement (***p***), transition load (***q***) and elastic stiffness (***E***) were extracted from the fit of the specimen loading curves (Table S2_4). Both in flexion and in extension, transition displacement showed similar and constant trend, except after nucleotomy (Friedman test: p= 0.26 in flexion, p= 0.68 in extension). The level of disc damage did not significantly impact transition load and elastic stiffness (Friedman test: ***q***: p= 0.93 in flexion, p= 0.65 in extension; ***E***: p=0.75 in flexion, p= 0.60 in extension) (Fig. S2_4, S2_5, S2_6).

Table S2_4– Transition displacement (p), transition load (q) and elastic stiffness (E) in the five discs damage conditions in flexion and in extension. The values were normalized against the intact condition. The mean between specimens is reported.

	p		***q***		***E***	
	Flexion	*Extension*	*Flexion*	*Extension*	*Flexion*	*Extension*
INT	1.00	1.00	1.00	1.00	1.00	1.00
2CUTS	0.77	0.95	0.77	1.18	0.96	1.02
4CUTS	0.75	0.88	0.75	0.93	0.97	1.03
SQR	0.67	0.88	0.69	0.96	1.02	1.06
NUCL	1.91	2.21	2.36	3.88	0.97	1.25

Note: The asterisks indicate where a statistically significant difference was found with respect to the intact (Nemenyi test) (*) p<0.05; (**) p<0.01; (***) p<0.001.

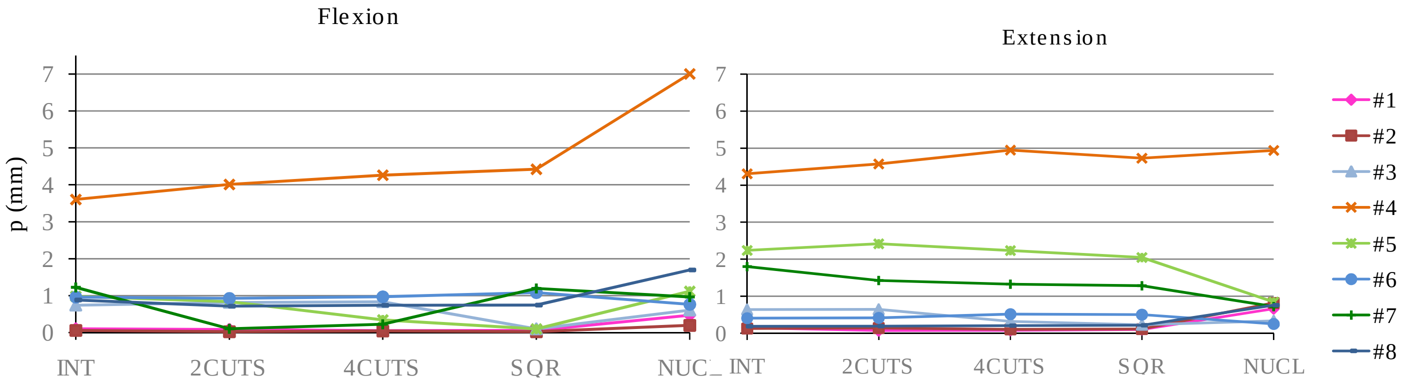

Fig. S2_4 – Transition displacement (p) in flexion and in extension in the different conditions, starting from intact (INT), and with progressive steps of incision (2CUTS, 4CUTS, SQR) in the annulus fibrosus, and after nucleotomy (NUCL). The median values over the five test repetitions are plotted for each of the eight specimens.

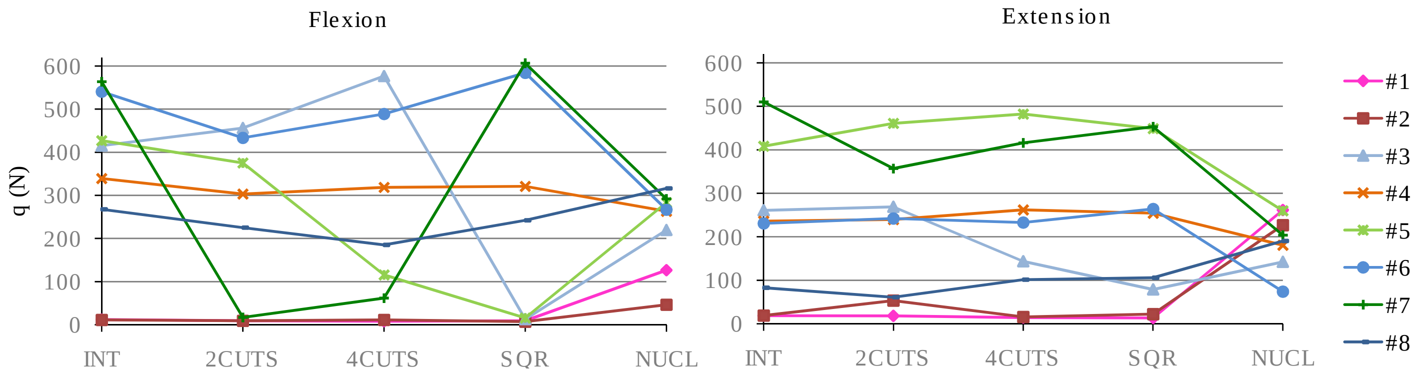

Fig. S2_5 – Transition load (q) in flexion and in extension in the different conditions, starting from intact (INT), and with progressive steps of incision (2CUTS, 4CUTS, SQR) in the annulus fibrosus, and after nucleotomy (NUCL). The median values over the five test repetitions are plotted for each of the eight specimens.

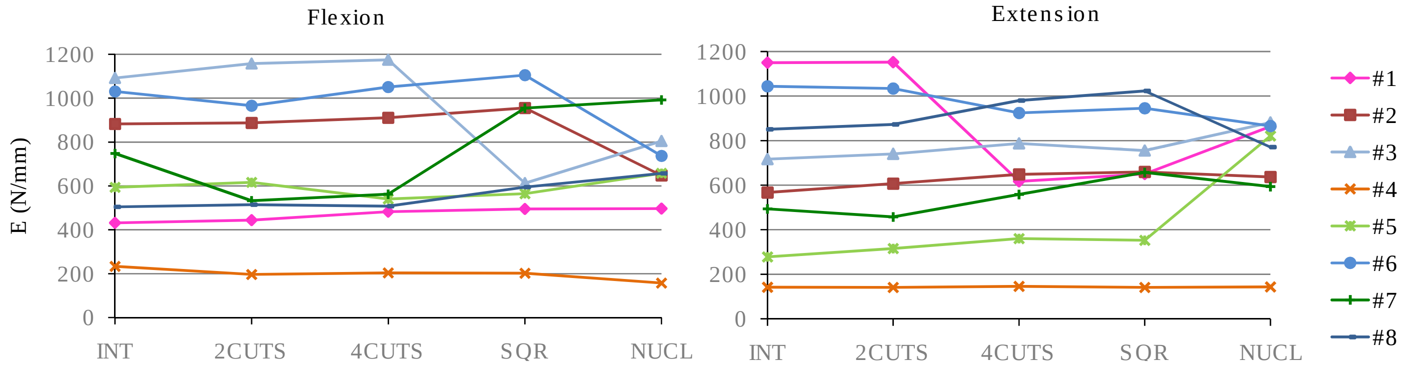

Fig. S2_6 – Elastic stiffness (**E**) in flexion and in extension in the different conditions, starting from intact (INT), and with progressive steps of incision (2CUTS, 4CUTS, SQR) in the annulus fibrosus, and after nucleotomy (NUCL). The median values over the five test repetitions are plotted for each of the eight specimens.

3.4 Strain distribution

The true maximum and minimum principal strains (ε_1 and ε_2) were extracted from all the tests at the peak load from the DIC correlations (Table S2_5). Both in flexion and in extension, the extreme strain values were in the disc while the strains in the vertebra were two orders of magnitude smaller. For both motions, the compressed part of the disc exhibited circumferential maximum and axial minimum principal strains whereas the stretched part of the disc showed circumferential maximum principal strains. In flexion, the maximum principal strain (ε_1) had a peak at mid-height of the disc, but did not follow any clear trend between disc conditions (Friedman test, p=0.79) (Fig. S2_7). With the increasing damage, the minimum principal strains (ε_2) located along the endplates in INT condition, migrated and concentrated covering the entire disc surface after NUCL condition (Friedman test, p= 0.06). In extension, most specimens showed constant maximum strains, and the extent of the lesion did not impact the maximum principal strain (ε_1) (Friedman test, p= 0.65) nor the minimum ones (ε_2) (p= 0.72).

Table S2_5 – Maximum and minimum principal strains ($\varepsilon_1$and ε_2) in flexion and in extension for the five conditions, normalized for each specimen with respect to the respective intact conditions. The mean of eight specimens is reported. A value larger than 1.00 indicates increased strains with respect to the intact condition.

	ε_1		ε_2	
	Flexion	*Extension*	*Flexion*	*Extension*
INT	1.00	1.00	1.00	1.00
2CUTS	1.01	1.07	0.99	0.98
4CUTS	1.00	1.15	0.93	1.02
SQR	1.02	1.08	0.99	0.98
NUCL	0.92	0.97	3.68	1.48

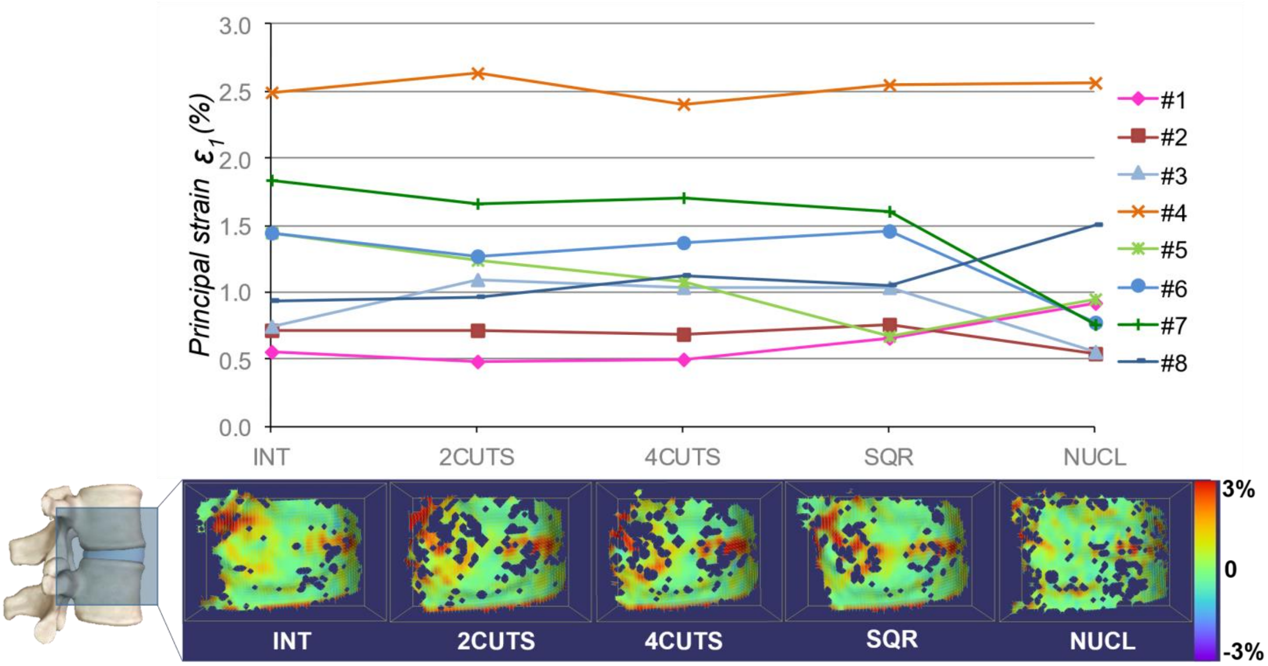

Fig. S2_7 – Top: maximum principal strain ($\mathcal{E}_1$), starting from intact (INT), and with progressive steps of incision (2CUTS, 4CUTS, SQR) in the annulus, and after nucleotomy (NUCL). The median over the five test repetitions is plotted for flexion for the eight specimens. Bottom: typical distribution of $\mathcal{E}_1$ on the disc surface in flexion. The dark spots are local non-correlated areas.

4 Discussion and Conclusion

The posterior disc height was not affected by annulus damages between INT and SQR conditions, but PDH decreased after nucleotomy, confirming the clinical observations (Varga et al., 2015) and the results reported by Showalter *et al* (Showalter et al., 2014). The sequential execution of disc damage did not significantly impact the spine biomechanics in terms of range of motion, stiffness parameters and strains.

Similar to this study, Kuroki *et al*., could not detect any significant difference in the flexion and extension range of motion after nucleotomy through a rectangular window in the annulus [9]. Lee *et al*. observed statistically significant differences between the range of motion in the intact and in the damaged disc, despite similar absolute values (Lee et al., 2018). The variations of stiffness of the present study are in agreement with Green *et* Adams, as they reported that collagen fibres do not need to be continuous to reinforce the annulus and that the fibre-matrix interactions make a large contribution to stiffness (Green et al., 1993). In addition, Michalek *et* Iatridis, concluded in 2012 that, the lack of changes in bending stiffness suggests that acute annular tears are not sufficient to induce off-axis motion and instability (Michalek and Iatridis, 2012). Few studies looked into the strain distribution on the disc surface under the same condition. Ruspi *et al*. found that different portions of the intervertebral disc were subjected to compression or tension with different orientation of the strains (Ruspi et al., 2017). The same was observed in this study: in the compressed side of the disc the minimum strains were axial, while circumferential maximum strains were located on the stretched side of the disc.

In conclusion, this study has shown that sequential damages of the annulus fibrosus does not significantly alter the spine biomechanics in terms of range of motion, stiffness, and strain distribution. The main effect caused by nucleotomy was the posterior disc height reduction due to the lack of support caused by the nucleus loss. The present findings therefore confirm the suitability of the model adopted in the main paper to simulate nucleotomy.

5 Additional References

Galbusera, F., van Rijsbergen, M., Ito, K., Huyghe, J.M., Brayda-Bruno, M., Wilke, H.-J., 2014. Ageing and degenerative changes of the intervertebral disc and their impact on spinal flexibility. Eur. Spine J. Off. Publ. Eur. Spine Soc. Eur. Spinal Deform. Soc. Eur. Sect. Cerv. Spine Res. Soc. 23 Suppl 3, S324-332. https://doi.org/10.1007/s00586-014-3203-4

Green, T.P., Adams, M.A., Dolan, P., 1993. Tensile properties of the annulus fibrosus II. Ultimate tensile strength and fatigue life. Eur. Spine J. Off. Publ. Eur. Spine Soc. Eur. Spinal Deform. Soc. Eur. Sect. Cerv. Spine Res. Soc. 2, 209–214. https://doi.org/10.1007/BF00299448

Kirkaldy-Willis, W.H., Farfan, H.F., 1982. Instability of the lumbar spine. Clin. Orthop. 110–123.

Kuroki, H., Goel, V.K., Holekamp, S.A., Ebraheim, N.A., Kubo, S., Tajima, N., 2004. Contributions of flexion-extension cyclic loads to the lumbar spinal segment stability following different discectomy procedures. Spine 29, E39-46. https://doi.org/10.1097/01.brs.0000106683.84600.e5

Lee, T., Lim, T.-H., Lee, S.-H., Kim, J.-H., Hong, J., 2018. Biomechanical function of a balloon nucleus pulposus replacement system: A human cadaveric spine study. J. Orthop. Res. Off. Publ. Orthop. Res. Soc. 36, 167–173. https://doi.org/10.1002/jor.23607

Michalek, A.J., Iatridis, J.C., 2012. Height and torsional stiffness are most sensitive to annular injury in large animal intervertebral discs. Spine J. 12, 425–432. https://doi.org/10.1016/j.spinee.2012.04.001

Ruspi, M.L., Palanca, M., Faldini, C., Cristofolini, L., 2017. Full-field in vitro investigation of hard and soft tissue strain in the spine by means of Digital Image Correlation. Muscles Ligaments Tendons J. 7, 538–545. https://doi.org/10.11138/mltj/2017.7.4.538

Showalter, B.L., Malhotra, N.R., Vresilovic, E.J., Elliott, D.M., 2014. Nucleotomy reduces the effects of cyclic compressive loading with unloaded recovery on human intervertebral discs. J. Biomech. 47, 2633–2640. https://doi.org/10.1016/j.jbiomech.2014.05.018

Tanaka, N., An, H.S., Lim, T.-H., Fujiwara, A., Jeon, C.-H., Haughton, V.M., 2001. The relationship between disc degeneration and flexibility of the lumbar spine. Spine J. 1, 47–56. https://doi.org/10.1016/S1529-9430(01)00006-7

Thompson, R.E., Pearcy, M.J., Barker, T.M., 2004. The mechanical effects of intervertebral disc lesions. Clin. Biomech. 19, 448–455. https://doi.org/10.1016/j.clinbiomech.2004.01.012

Thompson, R.E., Pearcy, M.J., Downing, K.J., Manthey, B.A., Parkinson, I.H., Fazzalari, N.L., 2000. Disc lesions and the mechanics of the intervertebral joint complex. Spine 25, 3026–3035. https://doi.org/10.1097/00007632-200012010-00010

Varga, P.P., Jakab, G., Bors, I.B., Lazary, A., Szövérfi, Z., 2015. Experiences with PMMA cement as a stand-alone intervertebral spacer. Orthop. 44, 1–8. https://doi.org/10.1007/s00132-014-3060-1
