# Supplementary material for: Biomechanical consequences of cement discoplasty: An in vitro study on thoraco-lumbar human spines
Source: Front Bioeng Biotechnol. 2022 Dec 2;10:1040695. doi: 10.3389/fbioe.2022.1040695 (PMC9755512; doi:10.3389/fbioe.2022.1040695)
Supplement: Supplementary file 2 [file DataSheet1.PDF]

*Supplementary Material #1*

# Additional methodological details and results

Appendix to the paper

**Biomechanical consequences of cement discoplasty: an *in vitro* study on thoraco-lumbar human spines**

## 1 Materials and Methods - Technical details of Data analysis

The parameters were extracted from the $6^{th}$ cycle of each test repetition for both directions of loading (Fig. 3). All measurements were compared for each specimen between the two conditions: nucleotomy (NUCL), and percutaneous cement discoplasty (PCD). In order to assess the changes of the height of neuroforamen, which is the main reason for performing PCD, the posterior disc height (PDH) was measured using the DIC images at the peak load: one point on each endplate was identified on the 3D profile in the posterior region of the disc, close to the neuroforamen. The points were aligned in the cranial-caudal direction. Their position was therefore tracked using DIC software. Measurements were performed three times for each disc condition (NUCL, PCD) and for each loading configuration (flexion, extension), and the mean was computed.

Applying singular value decomposition (SVD) on rigid bodies, the motions (translations and rotations) of each vertebra were computed from DIC images with a Matlab script (Morosato et al., 2019; Techens et al., 2020). The range of motion (ROM) was defined as the relative angle between the vertebra in the sagittal plane between the peak load and unloaded conditions.

Load-displacement data were smoothed using a median filter (over 30 data points) and the last cycle of the test was isolated. Then, its loading part was fitted by a continuous curve-fitting method to characterize the specimen stiffness (Tanaka et al., 2011). An exponential curve was applied to the toe region followed by a linear curve to the elastic region (Eq. 1).

$$F = \begin{cases} A\left(e^{B\delta} - 1\right) & \delta \leq p \\ E(\delta - p) + q & \delta > p \end{cases} \qquad (Eq. 1)$$

Where:

$F$ is the applied force
$\delta$ is the measured displacement
$A$, $B$, $E$, $p$, $q$ are specimen-specific parameters

The laxity (LZ) and elastic zones (EZ) were identified from load-displacement curves as respectively the region of large mobility and no loading, and the region where tissue stretched, characterizing the transition point in between (Fig. S1_1). Stiffness was characterized on the toe region by the parameters A, B and in particular their product A*B which describes the stiffness at the initial loading conditions. The elastic stiffness in the linear region starting at the transition point (p, q) is defined by the slope E of the curve.

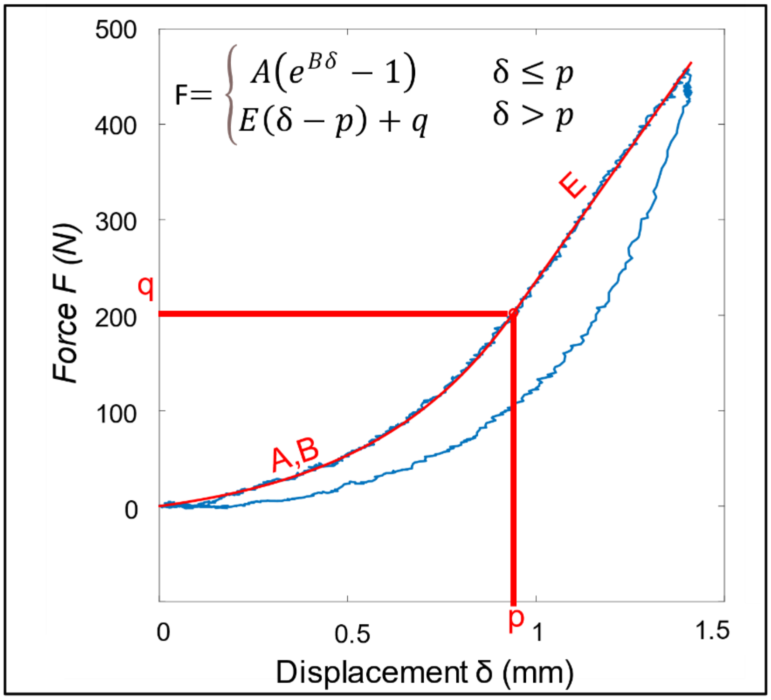


**Fig. S1_1** Typical load-displacement curve followed by the specimens in the majority of the tests (79/104): this was described with an exponential toe region, and linear elastic part which were fitted following Tanaka et al. model

In addition, the true principal strains over the specimen surface (vertebra and IVD) were measured at the peak load. In particular, the disc surface area was manually identified using the four dots on DIC images. The maximum and minimum true principal strains ($\varepsilon_1$ and $\varepsilon_2$) were analysed. Their median over the surface were computed, as well as their extreme values (defined as the 95%-percentile, to avoid local measurement artefacts).

# 2 Additional Results

## 2.1 Stiffness

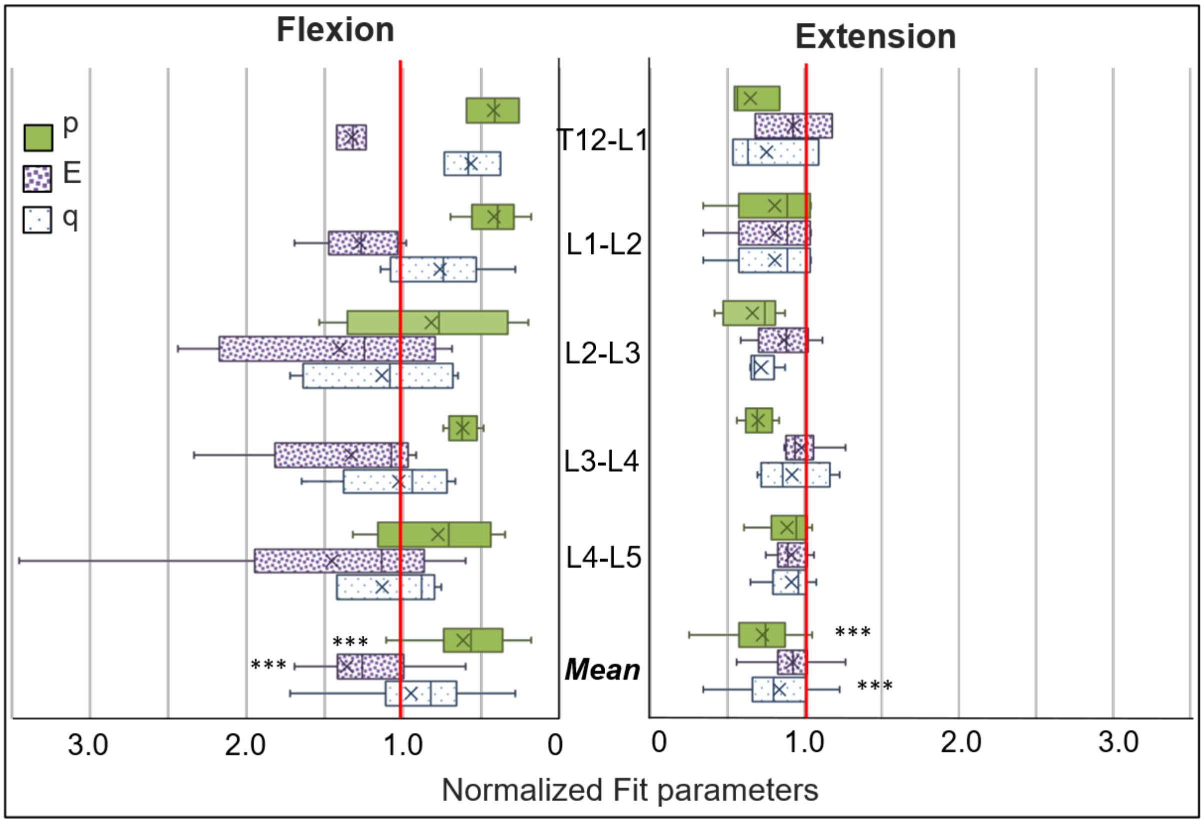


**Fig. S1_2** Stiffness parameters changes caused by cement discoplasty depending on the spine level. p: transition point displacement, E: elastic stiffness, q: transition point load. The values after discoplasty were normalized against the respective values after nucleotomy. A value above 1.00 means the parameter increased in discoplasty compared to the nucleotomy condition. A value smaller than 1.00 means discoplasty reduced that parameter. First quartile, median and third quartile are represented by lines. Mean is indicated by the cross and min and max values by the whiskers. Statistical significance (paired t-test, $p<0.001$) is designated by ***

## 2.2 Strain

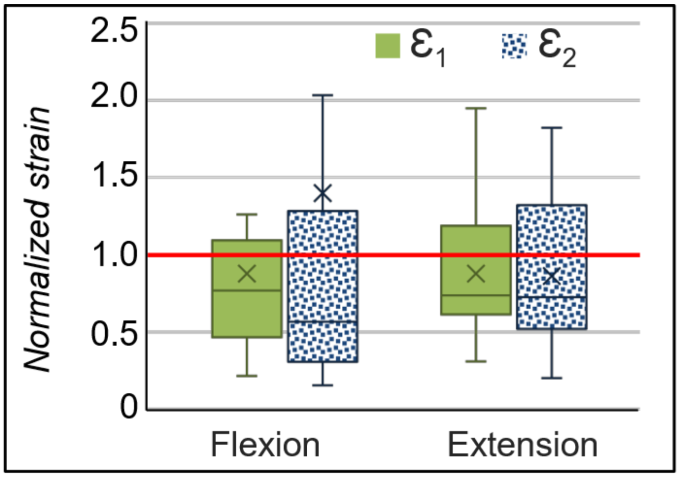


**Fig. S1_3** Median values of the maximum Ɛ1 and minimum Ɛ2 strains over the specimen surface measured at peak load in flexion and extension. The values after discoplasty were normalized against the respective values after nucleotomy. A value above 1.00 means the median strain increased in discoplasty compared to the nucleotomy condition. A value smaller than 1.00 means discoplasty reduced the strain compared to nucleotomy. First quartile, median and third quartile are represented by lines. Mean is indicated by the cross and min and max values by the whiskers

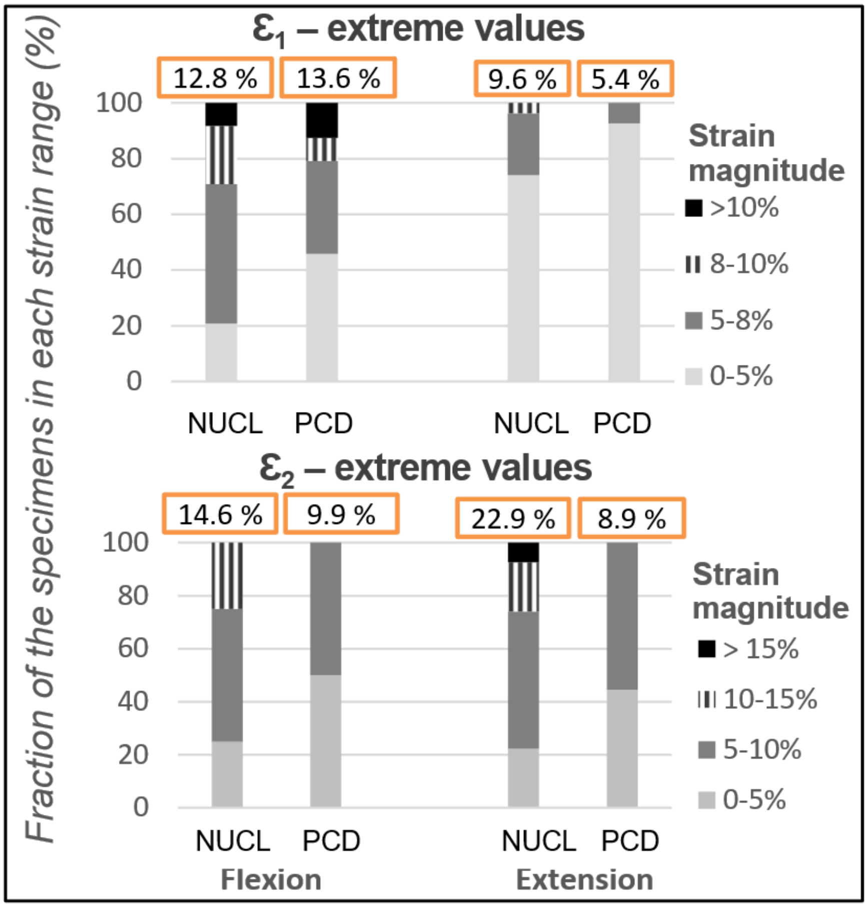


**Fig. S1_4** Analysis of the absolute extreme values of the maximum ($\varepsilon_1$) and minimum ($\varepsilon_2$) principal strains among the 27 specimens. For each disc condition (NUCL/PCD), the fraction of specimens falling in each strain range is presented, with the largest absolute strain value measured among the discs being displayed for each condition

## 2.3 Cement geometry visualisation and thickness measurement

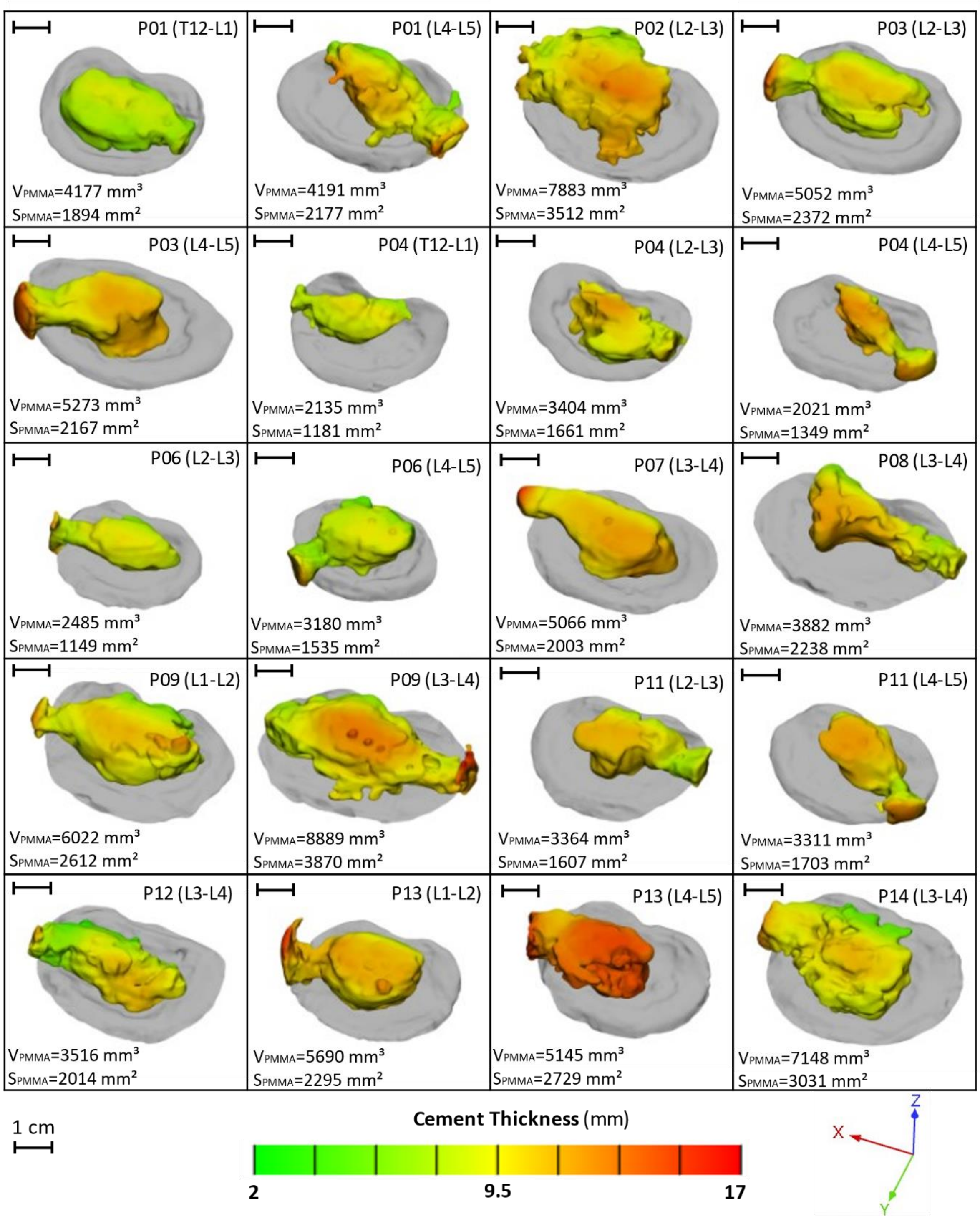


**Fig. S1_5** Visualization of the cement distribution over the caudal endplate geometry and thickness measurement. In the presented specimens no endplate perforation was observed. The mean±SD volume is 4591±1863 $mm^3$, and surface is 2155±726 $mm^2$. Thickness is represented by a 2-17 mm colourmap scale ranging from green to red. The xyz coordinate system defines the view

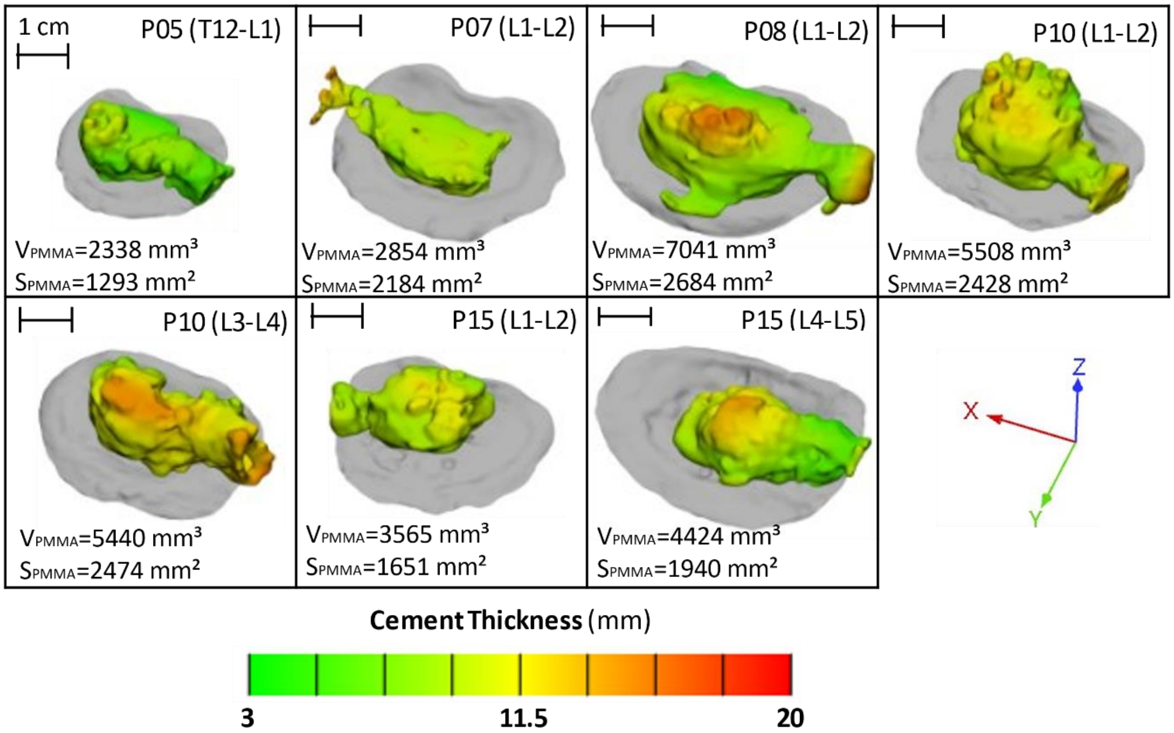


**Fig. S1_6** Visualization of the cement distribution over the caudal endplate geometry and thickness measurement. In the presented specimens, endplate perforation was observed. The mean±SD volume was 4453±1664 mm³, and surface was 2093±496 mm$^2$. Thickness is represented by a colourmap scale ranging from green to red. The xyz coordinate system defines the view

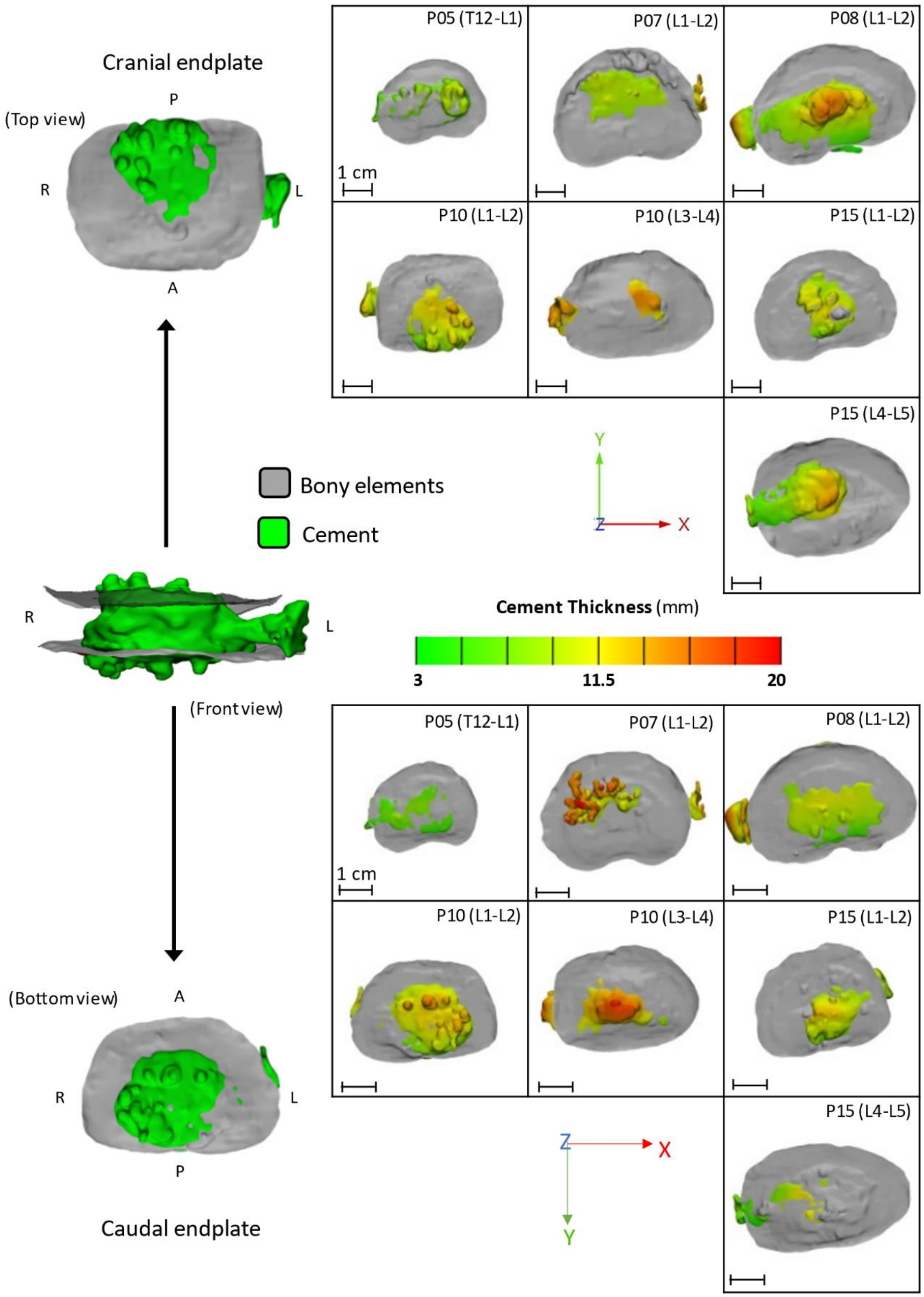


**Fig. S1_7** Visualization of the cement leakage geometry through the cranial (up) and caudal (down) endplate and thickness measurement. In the presented specimens, endplate perforation was observed. Thickness is represented by a colourmap scale ranging from green to red. The xyz coordinate system defines the view

**Table S1_1** - Summary of the biomechanical parameters for each loading configuration (flexion and extension) and disc condition (nucleotomy (NUCL) and percutaneous cement discoplasty (PCD)). The variation trend between absolute values of NUCL and PCD is indicated with increasing ⇑ and decreasing ⇓ arrows. The mean (SD) between specimens is reported

| | | Posterior disc height (mm)* | ROM (°) | Stiffness parameters | | | $\varepsilon_1$ (%) | | $\varepsilon_2$ (%) | |
|---|---|---|---|---|---|---|---|---|---|---|
| | | | | **p (mm)** | **q (N)** | **E (N/mm)** | **Mean (SD)** | **95-percentile** | **Mean (SD)** | **95-percentile** |
| **Flexion (n=24)** | **NUCL** | 5.5 (1.4) | 4.8 (1.2) | 3.3 (1.3) | 283 (76) | 313 (126) | 1.9 (0.7) | 6.9 (2.5) | -1.3 (1.0) | -7.6 (3.6) |
| | **PCD** | 7.3 (1.6) ⇑ | 3.5 (2.0) ⇓ | 2.0 (1.4) ⇓ | 259 (111) ⇓ | 400 (165) ⇑ | 1.4 (0.5) ⇓ | 5.8 (2.9) ⇓ | -0.7 (0.3) ⇓ | -5.4 (2.2) ⇓ |
| **Extension (n=27)** | **NUCL** | 5.8 (1.9) | 2.3 (1.0) | 2.2 (0.8) | 327 (77) | 629 (165) | 1.1 (0.5) | 4.5 (1.6) | -0.9 (0.6) | -8.6 (4.8) |
| | **PCD** | 7.5 (2.2) ⇑ | 2.2 (0.9) ⇓ | 1.6 (0.9) ⇓ | 269 (77) ⇓ | 577 (190) ⇓ | 0.9 (0.3) ⇓ | 3.1 (1.0) ⇓ | -0.6 (0.3) ⇓ | -5.1 (1.9) ⇓ |

* For both NUCL and PCD, the posterior disc height in extension is superior to flexion. Because n=24 specimens were averaged in flexion and n=27 in extension., both motions cannot be compared.

## 3 Additional References


Morosato, F., Traina, F., Cristofolini, L., 2019. Effect of different motor tasks on hip cup primary stability and on the strains in the periacetabular bone: An in vitro study. Clin. Biomech. 70, 137–145. https://doi.org/10.1016/j.clinbiomech.2019.08.005

Tanaka, M., Weisenbach, C., Miller, M., Kuxhaus, L., 2011. A Continuous Method to Compute Model Parameters for Soft Biological Materials. J. Biomech. Eng. 133, 074502. https://doi.org/10.1115/1.4004412

Techens, C., Palanca, M., Eltes, P.E., Lazary, A., Cristofolini, L., 2020. Testing the impact of discoplasty on the biomechanics of the intervertebral disc with simulated degeneration: an in vitro porcine study. Med. Eng. Phys. https://doi.org/10.1016/j.medengphy.2020.07.024
